# Regression methods for cost-effectiveness analysis with different censoring times or terminating events for survival time and costs

**DOI:** 10.1093/biomtc/ujag073

**Published:** 2026-05-12

**Authors:** Dingning Liu, Shuai Chen

**Affiliations:** Department of Statistics, University of California, Davis, CA 95616, United States; Division of Biostatistics, Department of Public Health Sciences, University of California, Davis, CA 95616, United States

**Keywords:** cost-effectiveness analysis, different censoring times, different terminating events, inverse-probability weighting, martingale process, survival analysis

## Abstract

Cost-effectiveness analysis (CEA) is crucial for evaluating new medical treatments. In many studies, both costs and effectiveness are censored. While standard survival analysis methods suit survival time, they cannot be directly applied to cumulative outcomes (e.g., costs or quality-adjusted lifetime) due to induced informative censoring. Additional challenges arise when costs and effectiveness have different terminating events or censoring times. Our motivating examples are two cardiovascular trials: MADIT-CRT and MADIT-II. In MADIT-CRT, effectiveness was defined as heart failure-free survival, while costs accumulated until death, leading to different terminating events for costs and effectiveness. Furthermore, subgroup identification for patient heterogeneity was also of interest. In MADIT-II, early stopping of cost collection for some patients complicated CEA. These examples highlight the need for methods handling different censoring structures while allowing covariate adjustment to improve efficiency and address imperfect randomization. Although some CEA methods have been proposed for different terminating events or censoring times, none provide covariate adjustment. We propose regression-based methods for CEA with different terminating events or censoring times, focusing on estimating the incremental cost-effectiveness ratio and the incremental net benefit. By incorporating covariates within a regression framework, our methods enable covariate adjustment and subgroup identification. Simulation studies demonstrate good finite-sample performance, and applications to the two motivating examples show that our approach provides practical tools for CEA under complex censoring mechanisms.

## 1 Introduction

As healthcare expenses continue to rise given limited resources, economic evaluations of new treatments have become crucial. When a new treatment has greater health benefits than its competitor (e.g., standard of care) but at higher costs, a decision must be made. There are two main summary statistics in cost-effectiveness analysis (CEA): the incremental cost-effectiveness ratio (ICER) and the incremental net benefit (INB). The ICER (Chaudhary and Stearns, 1996) represents the additional cost per unit of effectiveness gained from a new treatment, defined as $\Delta \mathrm{Cost}/\Delta \mathrm{Effect}$, where $\Delta \mathrm{Cost}$ and $\Delta \mathrm{Effect}$ are the differences in expected costs and effectiveness between the new and conventional treatments. Due to the problems associated with ratio statistics (e.g., skewness and potential infinite values), attention has recently shifted to the INB (Hoch et al., 2002). Given a decision maker’s willingness-to-pay (WTP) threshold $\lambda$ for an additional unit of effectiveness, the INB is defined as $\mathrm{INB}(\lambda )=\lambda \cdot \Delta \mathrm{Effect}-\Delta \mathrm{Cost}$. A positive INB or an ICER below $\lambda$ when both $\Delta \mathrm{Cost}$ and $\Delta \mathrm{Effect}$ are positive indicates that the new treatment is cost-effective. Two commonly used measures of treatment effectiveness are years of life (YOL) and quality-adjusted lifetime (QAL). The QAL accounts for both quality of life and survival time, offering a practical summary measure for evaluating treatment effects in chronic diseases (Gelber et al., 1989).

Censoring brings unique challenges in CEA. Two commonly used naive approaches for cost estimation are the complete-case (CC) method, which uses only uncensored data, and the all-data (AL) method, which ignores censoring status. Both are generally biased except in special cases. The AL method truncates survival time for censored patients at the censoring time, thereby underestimating costs. The CC method includes only complete observations, often from patients with shorter survival times. As a result, cost estimates may be biased depending on the association between survival and cost, as total costs are typically correlated with survival. Therefore, the AL and CC methods should be avoided when performing CEA with censored cost data. Additionally, traditional survival methods, such as the Kaplan–Meier (KM) estimator and Cox regression model, are not directly applicable to censored costs and QAL due to “induced informative censoring” (Lin et al., 1997). For example, patients with faster cost accumulation (or poorer quality of life) tend to incur greater costs (or lower QAL) both at the potential event time and censoring time, thereby violating the independent censoring assumption on the cost (or QAL) scale. To address this issue, many methods (Lin et al. 1997; Bang and Tsiatis, 2000; Zhao and Tsiatis 2000; Zhao and Tian, 2001) have been proposed to estimate the mean costs (or QAL) in the presence of censoring.

In CEA, confidence intervals (CIs) are often used to quantify uncertainty in ICER or INB. While INB CIs can be easily obtained via bootstrap or Wald-type methods (O’Brien and Briggs, 2002), the latter is unsuitable for ICER due to its skewed distribution and potential infinite values when the denominator is near zero. Alternatively, Fieller’s theorem (Fieller, 1954) can be adapted to derive an analytical formula for the CI of ICER under the large-sample normality assumption. Although bootstrap is commonly used and often favored for its superior coverage, Hwang (1995) and Jiang et al. (2000) demonstrated that both methods achieve first-order accuracy. Thus, the Fieller method provides a reliable and efficient approach for calculating ICER CIs. Whether using ICER or INB, estimation of between-treatment differences in cost and effectiveness, and their variances and covariance, is required to obtain CIs using the Fieller method for ICER or Wald-type methods for INB.

Different terminating events or censoring times for costs and effectiveness often arise in clinical studies, further complicating CEA. For example, the Multicenter Automatic Defibrillator Implantation Trial with Cardiac Resynchronization Therapy (MADIT-CRT) (Moss et al., 2009) assessed whether CRT with biventricular pacing would reduce the risk of death or heart failure (HF) in patients with mild cardiac symptoms. The terminating event is death or HF, whichever occurs first. However, since the interest is on healthcare costs accrued until death, the terminating events for costs and effectiveness may differ. Similar issues occur in cancer studies, where progression-free survival is a widely used endpoint, defined as the time to the first progression or death. Similarly, cost collection may end early due to funding constraints or billing inconsistencies. For example, MADIT-II (Moss et al., 2002) evaluated the survival benefit of a prophylactically implanted defibrillator versus conventional therapy in patients with prior myocardial infarction and advanced left ventricular dysfunction. In this study, cost data collection ended early for some patients, while survival data were available until study completion. To address these issues, methods have been proposed to estimate ICER and construct CIs for differently censored data (Wang and Zhao, 2006) and for different terminating events (Chen and Zhao, 2013). However, neither method accounts for covariates.

In observational studies, treatment assignment often depends on patient characteristics, making covariate adjustment essential to avoid confounding bias. In clinical trials, such adjustment improves efficiency and accounts for covariate imbalances resulting from imperfect randomization. Willan et al. (2005), Pullenayegum and Willan (2007), and Chen and Hoch (2022) proposed simple weighted and partitioned regression methods for censored cost-effectiveness (CE) data by extending Lin’s method (Lin, 2000). The partitioned method uses full cost and health histories to improve efficiency over the simple weighted method but depends on arbitrary partition time points. Wang and Zhao (2007) proposed a regression method for QAL integrating health history without specifying partition points. However, these regression methods are limited to data where costs and effectiveness share identical event and censoring times and do not accommodate differing terminating events or censoring mechanisms.

In this paper, we propose new regression methods and corresponding statistical inference procedure to address challenges in CEA with different terminating events or censoring times. The proposed methods flexibly allow different covariates for costs and effectiveness, while achieving efficiency by incorporating cost and health histories of all patients without relying on arbitrary partition points. Furthermore, our regression models can include covariate-treatment interactions for subgroup identification when CE is heterogeneous. Analytical formulas for the CIs of ICER and INB are obtained by deriving the covariances between costs and effectiveness, including survival time and QAL.

The remainder of the paper is organized as follows. In Section 2, we first briefly review existing methods and then describe the proposed regression methods to handle the challenges in analyzing censored CE data with different terminating events or different censoring times. The results of simulation studies and real data examples are given in Sections 3 and 4, respectively. Finally, we provide discussion and concluding remarks.

## 2 Methods

### 2.1 Notations and assumptions

For the *i*th subject in the study ($i=1,\ldots ,n$), we observe the follow-up time $X_i\equiv \mathrm{min}(T_i,C_i)$ and the event indicator $\Delta _i\equiv I(T_i\leqslant C_i)$, where $T_i$ is the survival time until the event of death, $C_i$ is the censoring time, and $I(\cdot )$ is the indicator function. Let $M_i(u)$ represent the costs accumulated to time *u*, then the total costs until death is $M_i(T_i)$, which is of interest but could be censored. For simplicity, we define $M_i=M_i(X_i)$ as the observed total costs until the last follow-up time. Survival time $T_i$ is a common measurement for effectiveness. For chronic diseases such as cardiovascular disease or cancer, using QAL as the effectiveness measure better reflects patients’ post-treatment quality of life. A patient’s health history is usually collected through surveys and then converted into a utility weight represented by $q_i(t)$ for the *i*th patient at time *t*. The *i*th patient’s QAL accrued up to time *u* is defined as the integration of utility weight over time, $Q_i(u)=\int _0^{u}q_i(t)dt$. Denote $Q_i=Q_i(X_i)$ as the observed total QAL and $Q_i(T_i)$ as the total QAL until death (potentially censored).

Let $V_i$ represent the *i*th patient’s outcome (e.g., $M_i(T_i)$, $Q_i(T_i)$, or $T_i$). Denote $\boldsymbol{Z}_i^V$ as the covariate vector for the *i*th patient and outcome *V*, including an intercept, treatment indicator $A_i$ (1 for new treatment, 0 for traditional treatment), covariates $\boldsymbol{U}^V_i=\lbrace U^V_{i1},\ldots ,U^V_{ip}\rbrace ^{^{\prime }}$, and treatment-covariate interactions $A_i\times \boldsymbol{U}^V_i$. Survival time, costs, and QAL are allowed to be correlated and depend on treatment and baseline covariates, as is typical in real-world settings. Assume that censoring time $C_i$ is independent of the survival $T_i$, cost history $M_i^H(X_i)=\lbrace M_i(u):u\leqslant X_i\rbrace$, and health history $Q_i^H(X_i)=\lbrace Q_i(u):u\leqslant X_i\rbrace$, conditional on $A_i$ and $\boldsymbol{U}_i$. This assumption is adopted in most survival data analyses. In addition, censoring makes it impossible to estimate costs over the entire health history without making additional assumptions beyond what is observed. Therefore, the CEA is restricted to a time horizon of *L* years to ensure valid statistical inference within the period supported by the data. While costs and survival accrue until death, the horizon *L* defines the specific evaluation window. Statistically, this is equivalent to analyzing restricted survival time $T_i^L = \min (T_i, L)$, where *L* is chosen so that an adequate number of subjects remain under observation at that time. For ease of notation, we suppress the superscript in $T_i^L$ throughout the paper.

The observed data for *n* subjects are independently identically distributed random variables $\lbrace X_i, \Delta _i, M_i(u), Q_i(u), u\leqslant X_i, \boldsymbol{Z}^V_i, i=1,\ldots ,n \rbrace$. The linear regression model takes the form


(1) \begin{eqnarray*}
E(V_i|\boldsymbol{Z}^V_i)=\beta _{V,0} +\beta _{V,A} A_i +\boldsymbol{\beta }^{^{\prime }}_{V,\boldsymbol{U}} \boldsymbol{U}^V_i+\boldsymbol{\beta }^{^{\prime }}_{V,A\boldsymbol{U}} A_i\boldsymbol{U}^V_i=\boldsymbol{\beta }_V^{^{\prime }} \boldsymbol{Z}^V_i,
\end{eqnarray*}

where $\boldsymbol{\beta }_V=(\beta _{V,0},\beta _{V,A},\boldsymbol{\beta }^{^{\prime }}_{V,\boldsymbol{U}},\boldsymbol{\beta }^{^{\prime }}_{V,A\boldsymbol{U}})^{^{\prime }}$ is the regression coefficients for outcome *V*. We will estimate $\boldsymbol{\beta }_V$ and its variance using the observed censored data with different terminating events or censoring times, and make inferences for ICER and INB while adjusting for covariates and/or evaluating heterogeneous CE across covariate groups.

### 2.2 Review of existing regression methods for a single censored outcome

Lin (2000) proposed a simple inverse-probability weighting (SW) estimator for $\boldsymbol{\beta }_V$ in Model (1):


(2) \begin{eqnarray*}
\hat{\boldsymbol{\beta}}_V^{\mathrm{SW}}=\left\{\sum_{i=1}^n \frac{\Delta_i}{\widehat{K}\left(T_i\right)} \boldsymbol{Z}_i^{V \otimes 2}\right\}^{-1}\left\{\sum_{i=1}^n \frac{\Delta_i}{\widehat{K}\left(T_i\right)} V_i \boldsymbol{Z}_i^V\right\},
\end{eqnarray*}

where $\boldsymbol{a}^{\otimes 2}=\boldsymbol{a} \boldsymbol{a}^{\prime}$ and $\boldsymbol{a} \otimes \boldsymbol{b}=\boldsymbol{a} \boldsymbol{b}^{\prime}$ for vectors $\boldsymbol {a}$ and $\boldsymbol {b}$. $\widehat{K}(u)$ is the KM estimator for the survival function of censoring time variable *C* at time *u*, $K(u)=\mathrm{Pr}(C> u)$, using ($X_i, \Delta _i, i=1,\ldots , n$). Note that covariate-dependent censoring can also be accommodated by estimating $K(u)$ using a Cox model (Lin, 2000). The SW approach is not efficient, as it only includes outcome *V* from uncensored patients. When cost and health histories are available, utilizing information from all observations can achieve higher estimating efficiency. Wang and Zhao (2007) proposed an improved estimator (IMP) for QAL by incorporating health history, which can also be applied to costs. The estimator is $\hat{\boldsymbol{\beta }}^{\mathrm{IMP}}_{V}=({\bf B}^V_{1})^{-1}{\bf B}^V_{2}$, where


(3) \begin{eqnarray*}
{\bf B}^V_{1}&=&\sum _{i=1}^n\left\lbrace \frac{\Delta _i}{\widehat{K}(T_i)}+\int _0^{L}\frac{\mathrm{d} N_i^C(u)}{\widehat{K}(u)} \right\rbrace {\boldsymbol{Z}^{V}_{i}}^{\otimes 2}\\&&-\sum _{i=1}^n \int _0^L\frac{\sum _{j=1}^n Y_j(u){\boldsymbol{Z}^{V}_{j}}^{\otimes 2}}{\widehat{K}(u)Y(u)}\mathrm{d}N_i^C(u), \\{\bf B}^{V}_{2}&=&\sum _{i=1}^n\left\lbrace \frac{\Delta _i V_i}{\widehat{K}(T_i)}+\int _0^{L}\frac{V_i(u)}{\widehat{K}(u)}\mathrm{d} N_i^C(u) \right\rbrace {\boldsymbol{Z}^{V}_{i}}\\&&-\sum _{i=1}^n \int _0^L\frac{\sum _{j=1}^n V_j(u)Y_j(u){\boldsymbol{Z}^{V}_{j}}}{\widehat{K}(u)Y(u)}\mathrm{d}N_i^C(u),
\end{eqnarray*}

$N^C(u)=\sum _{i=1}^n N_i^C(u)=\sum _{i=1}^n I(X_i\leqslant u,\Delta _i=0)$ , and $Y(u)=\sum _{i=1}^n Y_i(u)=\sum _{i=1}^n I(X_i\geqslant u)$. Denote $D_i(\boldsymbol{\beta }_V)={\boldsymbol{Z}^{V}_{i}}(V_i-\boldsymbol{\beta }_V^{^{\prime }} {\boldsymbol{Z}^{V}_{i}})$ and $D_i(\boldsymbol{\beta }_V,u)=\boldsymbol{Z}^{V}_{i}(V_i(u)-\boldsymbol{\beta }_V^{^{\prime }} \boldsymbol{Z}^{V}_{i})$. The asymptotic variance-covariance matrix of the IMP estimator can be estimated by


(4) \begin{eqnarray*}
\widehat{\mathrm{Var}}(\hat{\boldsymbol{\beta }}^{\mathrm{IMP}}_{V})=\frac{1}{n} (\hat{I}^{V}_{0})^{-1} {\hat{I}^{V}_{1}}(\hat{I}^{V}_{0})^{-1},
\end{eqnarray*}

where $\hat{I}^{V}_{0}=\frac{1}{n}\sum _{i=1}^n{\boldsymbol{Z}^{V}_{i}}^{\otimes 2}$,


(5) \begin{eqnarray*}
\hat{I}_1^V&= & \frac{1}{n} \sum_{i=1}^n \frac{\Delta_i}{\widehat{K}\left(T_i\right)}\left\{D_i\left(\hat{\boldsymbol{\beta}}_V^{\mathrm{IMP}}\right)\right\}^{\otimes 2} \\& &+\widehat{J}\left\{D\left(\hat{\boldsymbol{\beta}}_V^{\mathrm{IMP}}\right) \otimes D\left(\hat{\boldsymbol{\beta}}_V^{\mathrm{IMP}}\right)\right\}\\&&+\widehat{J}\left\{D\left(\hat{\boldsymbol{\beta}}_V^{\mathrm{IMP}}, u\right) \otimes D\left(\hat{\boldsymbol{\beta}}_V^{\mathrm{IMP}}, u\right)\right\} \\&& -\widehat{J}\left\{D\left(\hat{\boldsymbol{\beta}}_V^{\mathrm{IMP}}\right) \otimes D\left(\hat{\boldsymbol{\beta}}_V^{\mathrm{IMP}}, u\right)\right\}\\&&-\widehat{J}\left\{D\left(\hat{\boldsymbol{\beta}}_V^{\mathrm{IMP}}, u\right) \otimes D\left(\hat{\boldsymbol{\beta}}_V^{\mathrm{IMP}}\right)\right\},
\end{eqnarray*}

\begin{eqnarray*}
\widehat{J}(X \otimes Y)=\frac{1}{n} \int _0^L\left\lbrace \widehat{G}(X \otimes Y, u)-\widehat{G}(X, u) \otimes \widehat{G}(Y, u)\right\rbrace \frac{\mathrm{d} N^C(u)}{\widehat{K}(u)^2},
\end{eqnarray*}

$\widehat{G}(W,u)=\sum _{i=1}^n \lbrace \Delta _i/\widehat{K}(T_i)\rbrace W_i I(T_i\geqslant u)/\lbrace n\widehat{S}(u)\rbrace$ , and $\widehat{S}(u)$ is the KM estimator for survival distribution of $T_i$ at time *u*, $S(u)=\mathrm{Pr}(T> u)$, using data ($X_i, \Delta _i, i=1,\ldots , n$). When $\widehat{J}$ includes the term $D(\hat{\boldsymbol{\beta }}^{\mathrm{IMP}}_{V},u)$, in which $V_i(u)$ is the accumulated outcome *V* at time *u* for all patients, $\widehat{G}$ should be replaced by $\widehat{G}^{\star }(W,u)=\sum _{i=1}^n W_i Y_i(u)/Y(u)$. This adjustment utilizes historical information from censored patients, while $\widehat{G}$ only incorporates uncensored patients (i.e., the summand in $\widehat{G}$ is 0 for censored patients with $\Delta _i=0$). The estimated variance-covariance matrix for the SW estimator follows the same formula as in Equation (4), with $\hat{\boldsymbol{\beta }}^{\mathrm{IMP}}_{V}$ replaced by $\hat{\boldsymbol {\beta }}^{\mathrm{SW}}_{V}$, and includes only the first two terms of Equation (5).

### 2.3 Regression methods for different terminating events for survival time and costs

In this section, we propose new regression methods for CEA by extending the SW and IMP estimators to handle different terminating events for survival time and costs. The *i*th patient may experience a treatment failure event (e.g., heart failure or tumor progression) before death at time $T^{\mathrm{Event}}_i$, which may depend on treatment and baseline covariates and is allowed to correlate with survival time, costs, and QAL. We define the event-free survival time as $T_i^\mathrm{F} \equiv \mathrm{min}(T_i,T^{\mathrm{Event}}_i)$, where “F” denotes “free” (i.e., free from treatment failure event and death). For instance, if the treatment failure event is heart failure, $T_i^\mathrm{F}$ denotes the heart failure-free survival time. We assume that the censoring time $C_i$ is independent of $T^{\mathrm{Event}}_i$, possibly conditional on treatment $A_i$ and covariates $\boldsymbol{U}_i$. Denote $\Delta _i^\mathrm{F} \equiv I(T_i^\mathrm{F}\leqslant C_i)$ as the event-free survival indicator, $X_i^\mathrm{F} \equiv \mathrm{min}(T_i^\mathrm{F},C_i)$ as the event-free follow-up time, $Q_i^\mathrm{F}(u)=\int _0^{u}q_i(t)dt$ as the event-free QAL accrued until time *u*, and $Q_i^\mathrm{F}=Q_i(X_i^\mathrm{F})$ as the observed total event-free QAL. The restricted time horizon *L* is also applied to the event-free survival time, redefined as $T_i^\mathrm{FL}=\mathrm{min}(T_i^\mathrm{F},L)$, with the superscript *L* in $T_i^\mathrm{FL}$ omitted for the rest of the paper. Since costs are collected until death, the SW and IMP methods can be applied directly using Equations (2) and (3). However, adjustments are needed for event-free survival time and event-free QAL, and their covariances with costs accrued until death.

#### 2.3.1 The simple weighted approach for event-free survival time and event-free QAL

We extend the method of Chen and Zhao (2013) to allow covariate adjustment by modifying Equation (2) to estimate the SW regression coefficients for event-free effectiveness, $\hat{\boldsymbol{\beta }}^{\mathrm{SW}}_{V^\mathrm{F}}$, where $V^\mathrm{F}$ denotes event-free survival time $(T^\mathrm{F})$ or event-free QAL $(Q^\mathrm{F})$. Replacing *V* with $V^\mathrm{F}$, $T_i$ with $T_i^\mathrm{F}$, $\Delta _i$ with $\Delta _i^\mathrm{F}$, and $\widehat{K}(u)$ with $\widehat{K}^\mathrm{F}(u)$ in Equation (2), we obtain $\hat{\boldsymbol{\beta }}^{\mathrm{SW}}_{V^\mathrm{F}}=\left\lbrace \sum _{i=1}^n\frac{\Delta ^\mathrm{F}_i}{\widehat{K}^\mathrm{F}(T^\mathrm{F}_i)}{\boldsymbol{Z}^{V^\mathrm{F}}_{i}}^{\otimes 2}\right\rbrace ^{-1}\left\lbrace \sum _{i=1}^n\frac{\Delta ^\mathrm{F}_i}{\widehat{K}^\mathrm{F}(T^\mathrm{F}_i)}V^\mathrm{F}_i \boldsymbol{Z}^{V^\mathrm{F}}_{i}\right\rbrace$, where $\widehat{K}^\mathrm{F}(u)$ is the KM estimator for $K^\mathrm{F}(u)=\mathrm{Pr}(C> u)$, the survival function of censoring time *C* at time *u*, using data ($X^\mathrm{F}_i, \Delta ^\mathrm{F}_i, i=1,\ldots , n$). The estimated variance matrix of $\hat{\boldsymbol{\beta }}^{\mathrm{SW}}_{V^\mathrm{F}}$ can be adapted from Equation (4), with full details provided in Web Appendix A.1.1.

#### 2.3.2 The improved approach for event-free QAL using health history

Since QAL is derived from the available health history, we extend the method of Wang and Zhao (2007) to obtain an IMP estimator for the regression coefficients of event-free QAL ($Q^\mathrm{F}$). Specifically, $\hat{\boldsymbol{\beta }}^{\mathrm{IMP}}_{Q^\mathrm{F}}=({\bf B}^{Q^\mathrm{F}}_{1})^{-1}{\bf B}^{Q^\mathrm{F}}_{2}$, where ${\bf B}^{Q^\mathrm{F}}_{1}$ and ${\bf B}^{Q^\mathrm{F}}_{2}$ are obtained from Equation (3), adding the superscript $\mathrm{F}$ to $T_i$, $\Delta _i$, *Y*, $N_i(u)$, and $\widehat{K}(u)$ to reflect the event-free quantities. The explicit formula for $\hat{\boldsymbol{\beta }}^{\mathrm{IMP}}_{Q^\mathrm{F}}$ and its asymptotic variance estimator are provided in Web Appendix A.1.1.

#### 2.3.3 ICER and corresponding CI

We first use event-free QAL as the effectiveness measure. With different terminating events, the ICER is defined as the additional mean costs for a new treatment saving one year of event-free QAL, that is,


(6) \begin{eqnarray*}
\gamma =\frac{\Delta _M}{\Delta _{Q^\mathrm{F}}},
\end{eqnarray*}

where $\Delta _M$ and $\Delta _{Q^\mathrm{F}}$ are the covariate-adjusted mean differences in costs and event-free QAL between two treatment groups, respectively. When there is no covariates-treatment interaction in regression model (1) (i.e., the treatment effects are not heterogeneous), $\Delta _M$ and $\Delta _{Q^\mathrm{F}}$ can be estimated using the coefficient estimator for the treatment indicator. That is, $\hat{\Delta }_M=\hat{\beta }_{M,A}$ and $\hat{\Delta }_{Q^\mathrm{F}}=\hat{\beta }_{Q^\mathrm{F},A}$ using the SW or the IMP method. When there are interactions between covariates and treatment, CE becomes heterogeneous depending on the covariates. A detailed discussion of this is provided in Section 2.6.

Since $\hat{\boldsymbol{\beta }}_{M}$ and $\hat{\boldsymbol{\beta }}_{Q^\mathrm{F}}$ are asymptotically normally distributed, $\hat{\Delta }_M$ and $\hat{\Delta }_{Q^\mathrm{F}}$ are also asymptotically normal. Using Fieller’s Theorem (Fieller, 1954), the $100(1-2\alpha )\%$ confidence limits for the ICER $\gamma$ are given by


(7) \begin{eqnarray*}
&&\left[ \hat{\Delta}_M \hat{\Delta}_{Q^\mathrm{F}}-z_{\alpha}^2 s_{M Q^\mathrm{F}}\pm \{(\hat{\Delta}_M \hat{\Delta}_{Q^\mathrm{F}}-z_{\alpha}^2 s_{M Q^\mathrm{F}})^2 \right. \\&&\left. -(\hat{\Delta}_M^2-z_{\alpha}^2 s_{M})(\hat{\Delta}_{Q^\mathrm{F}}^2-z_{\alpha}^2 s_{Q^\mathrm{F}})\}^{1/2} \right] \Big/ (\hat{\Delta}_{Q^\mathrm{F}}^2-z_{\alpha}^2 s_{Q^\mathrm{F}}),
\end{eqnarray*}

where $z_{\alpha }$ is the $100(1-\alpha )$ percentile of the standard normal distribution, $s_{M}$ and $s_{Q^\mathrm{F}}$ are the estimated variances of $\hat{\Delta }_M$ and $\hat{\Delta }_{Q^\mathrm{F}}$, which are provided in Section 2.2 and Web Appendix A.1.1. $s_{M Q^\mathrm{F}}$ is the covariance between $\hat{\Delta }_M$ and $\hat{\Delta }_{Q^\mathrm{F}}$. In Web Appendix A.1.2, we use martingale and counting process theory to derive the covariances between the regression coefficients of costs and effectiveness (event-free survival time or event-free QAL) for the SW or IMP methods. Under the IMP method, the covariance can be estimated by $\widehat{\mathrm{Cov}}(\hat{\boldsymbol{\beta }}^{\mathrm{IMP}}_{M},\hat{\boldsymbol{\beta }}^{\mathrm{IMP}}_{Q^\mathrm{F}})=\frac{1}{n} (\hat{I}^M_{0})^{-1} \hat{I}_1^{MQ^\mathrm{F}} (\hat{I}^{Q^\mathrm{F}}_{0})^{-1}$, where $\hat{I}^M_{0}=\frac{1}{n}\sum _{i=1}^n {\boldsymbol{Z}^{M}_{i}}^{\otimes 2}$, $\hat{I}_{0}^{Q^\mathrm{F}}=\frac{1}{n}\sum _{i=1}^n {\boldsymbol{Z}^{Q^\mathrm{F}}_{i}}^{\otimes 2}$,


(8) \begin{eqnarray*}
&& \hat{I}_1^{M Q^{\mathrm{F}}}= \frac{1}{n} \sum_{i=1}^n \frac{\Delta_i D_i\left(\hat{\boldsymbol{\beta}}_{Q^{\mathrm{F}}}^{\mathrm{IMP}}\right) \otimes D_i\left(\hat{\boldsymbol{\beta}}_M^{\mathrm{IMP}}\right)}{\hat{K}\left(T_i\right)}\\&&-\frac{1}{n^2} \sum_{i=1}^n \frac{\Delta_i^{\mathrm{F}} D_i\left(\hat{\boldsymbol{\beta}}_{Q^{\mathrm{F}}}^{\mathrm{IMP}}\right)}{\hat{K}^{\mathrm{F}}\left(T_i^{\mathrm{F}}\right)} \otimes \sum_{i=1}^n \frac{\Delta_i D_i\left(\hat{\boldsymbol{\beta}}_M^{\mathrm{IMP}}\right)}{\hat{K}\left(T_i\right)} \\& &+\widehat{J}^{\mathrm{F}}\left\{D\left(\hat{\boldsymbol{\beta}}_{Q^{\mathrm{F}}}^{\mathrm{IMP}}\right) \otimes D\left(\hat{\boldsymbol{\beta}}_M^{\mathrm{IMP}}\right)\right\}\\&&-\widehat{J}^{\mathrm{F}}\left\{D\left(\hat{\boldsymbol{\beta}}_{Q^{\mathrm{F}}}^{\mathrm{IMP}}\right) \otimes D\left(\hat{\boldsymbol{\beta}}_M^{\mathrm{IMP}}, u\right)\right\} \\& &-\widehat{J}^{\mathrm{F}}\left\{D\left(\hat{\boldsymbol{\beta}}_{Q^{\mathrm{F}}}^{\mathrm{IMP}}, u\right) \otimes D\left(\hat{\boldsymbol{\beta}}_M^{\mathrm{IMP}}\right)\right\}\\&&+\widehat{J}^{\mathrm{F}}\left\{D\left(\hat{\boldsymbol{\beta}}_{Q^{\mathrm{F}}}^{\mathrm{IMP}}, u\right) \otimes D\left(\hat{\boldsymbol{\beta}}_M^{\mathrm{IMP}}, u\right)\right\},
\end{eqnarray*}

\begin{eqnarray*}
\widehat{J}^{\mathrm{F}}(X \otimes Y)=\frac{1}{n} \int _0^L\lbrace \widehat{G}^\mathrm{F}(X \otimes Y, u)-\widehat{G}^\mathrm{F}(X, u) \otimes \widehat{G}^\mathrm{F}(Y, u)\rbrace \frac{\mathrm{d} N^\mathrm{F}(u)}{\widehat{K}^\mathrm{F}(u)^2},
\end{eqnarray*}

$\widehat{G}^\mathrm{F}(W,u)=\frac{1}{n}\frac{1}{\widehat{S}^\mathrm{F}(u)}\sum _{i=1}^n \frac{\Delta ^\mathrm{F}_i W_i I(T^\mathrm{F}_i\geqslant u)}{\widehat{K}^\mathrm{F}(T_i^\mathrm{F})}$ , $N^\mathrm{F}(u)=\sum _{i=1}^n N_i^\mathrm{F}(u)=\sum _{i=1}^n I(X_i^\mathrm{F}\leqslant u,\Delta _i^\mathrm{F}=0)$, and $\widehat{S}^\mathrm{F}(u)$ is the KM estimator for the survival function of $T_i^\mathrm{F}$ at time *u*, $S^\mathrm{F}(u)=\mathrm{Pr}(T_i^\mathrm{F}> u)$, using data ($X^\mathrm{F}_i, \Delta ^\mathrm{F}_i, i=1,\ldots , n$). Similar to $\widehat{J}$, if $\widehat{J}^\mathrm{F}$ involves $D(\hat{\boldsymbol{\beta }}^{\mathrm{IMP}}_{V},u)$, $\widehat{G}^\mathrm{F}$ is replaced by $\widehat{G}^{\mathrm{F}\star }(W,u)=\frac{\sum _{i=1}^n W_i Y_i^\mathrm{F}(u)}{Y^\mathrm{F}(u)}$ to utilize historical data from all patients, while $\widehat{G}^\mathrm{F}$ only includes uncensored patients. When both costs and event-free QAL are estimated using the SW method, the covariance estimator includes only the first three terms in Equation (8).

Fieller’s method can yield unbounded CIs under certain conditions. Equation (7) is obtained by solving a quadratic inequality, and the term inside the square root corresponds to the associated discriminant. When both the discriminant and the denominator are positive, the CI is a finite interval between the two roots. When the discriminant is positive but the denominator is negative, the confidence set is unbounded and forms a disjoint interval given by the complement of the finite interval between the two roots. When the discriminant is negative, the confidence set is the entire real line if the denominator is negative or empty if the denominator is positive (Fan and Zhou, 2007). The unbounded or empty confidence sets typically arise when the estimated mean effectiveness difference is near zero, causing high ICER uncertainty (Hwang, 1995). When the true effectiveness difference is zero or near zero, the ICER is ill-defined, and such confidence sets reflect the intrinsic nonidentifiability of the ratio. Instead of relying on ICER CIs alone, uncertainty can be better summarized on the CE plane by examining the joint distribution of incremental costs and effects using asymptotic bivariate normal approximations or resampling-based methods.

The ICER and its CI using event-free YOL (i.e., event-free survival time) as the effectiveness measure can be estimated similarly to event-free QAL. Since YOL does not have a longitudinal history, the SW method suffices for its estimation. When costs are estimated using the IMP method, the covariance estimator involves only the first four terms in Equation (8); when both costs and event-free YOL are estimated via SW method, only the first three terms are included. See Web Appendix A.1.2 for the proof and more details.

### 2.4 Regression methods for different censoring times for survival time and costs

In many clinical studies, cost collection often ends before survival data collection. A common but naive approach, censoring survival time at the cost censoring time, is inefficient as it discards available information. In this section, we propose new regression methods for CEA to accommodate early censored costs. Let $C_{0i}$ be the censoring time for cost collection. For some subjects, $C_{0i}=C_i$. However, some cost data are censored earlier than survival data, such that $C_{0i}< C_i$. Denote the observed follow-up time of cost collection $X_{0i} \equiv \mathrm{min}(T_i,C_{0i})$, the indicator $\Delta _{0i} \equiv I(T_i\leqslant C_{0i})$, the accumulated costs at time *u*, $M_{0i}(u)$, and the observed total costs $M_{0i}=M(X_{0i})$. We assume that $C_{0i}$ is independent of the survival time $T_i$, cost history process, and health history process, conditional on treatment $A_i$ and covariates $\boldsymbol{U}_i$. The SW and IMP regression estimators for survival time and QAL can be obtained by Equations (2) and (3). However, adjustments are needed in these approaches for early-censored costs.

#### 2.4.1 The simple weighted approach for early-censored costs

The SW regression coefficient estimator for early-censored costs $\hat{\boldsymbol{\beta }}^{\mathrm{SW}}_{M_0}$ can be obtained by extending the method of Wang and Zhao (2006) to incorporate covariates. By adapting Equation (2) with $M_0$ as the outcome, and replacing $\Delta _{i}$ with $\Delta _{0i}$, and $\widehat{K}(u)$ with $\widehat{K}_0(u)$, we obtain $\hat{\boldsymbol {\beta }}^{\mathrm{SW}}_{M_0}=\left\lbrace \sum _{i=1}^n\frac{\Delta _{0i}}{\widehat{K}_0(T_i)}{\boldsymbol{Z}^{M_0}_{i}}^{\otimes 2}\right\rbrace ^{-1}\left\lbrace \sum _{i=1}^n\frac{\Delta _{0i}}{\widehat{K}_0(T_i)}M_{0i} \boldsymbol{Z}^{M_0}_{i}\right\rbrace$, where $\widehat{K}_0(u)$ is the KM estimator for the survival function of $C_0$ at time *u*, $K_0(u)=\mathrm{Pr}(C_0> u)$, using data ($X_{0i}, \Delta _{0i}, i=1,\ldots , n$). The corresponding variance estimator is provided in Web Appendix A.2.1.

#### 2.4.2 The improved approach for early-censored costs using cost history

When cost history is available, the IMP regression coefficient estimator is given by $\hat{\boldsymbol{\beta }}^{\mathrm{IMP}}_{M_0}=({\bf B}_1^{M_0})^{-1}{\bf B}_2^{M_0}$, where ${\bf B}_1^{M_0}$ and ${\bf B}_2^{M_0}$ correspond to ${\bf B}^{V}_{1}$ and ${\bf B}^{V}_{2}$ in Equation (3), with the subscript 0 added to $\Delta$, *C, Y*, and $\widehat{K}$ to account for early censoring of cost data. The explicit formula for $\hat{\boldsymbol{\beta }}^{\mathrm{IMP}}_{M_0}$ and its asymptotic variance estimator are provided in Web Appendix A.2.1.

#### 2.4.3 ICER and corresponding CI

To obtain the ICER and its CI for data with different censoring times using QAL as the effectiveness measure, we can use Equations (6) and (7) by replacing $\Delta _M$ with $\Delta _{M_0}$, $\Delta _{Q^\mathrm{F}}$ with $\Delta _{Q}$, $\boldsymbol{\beta }_{M}$ with $\boldsymbol{\beta }_{M_0}$, $\boldsymbol{\beta }_{Q^\mathrm{F}}$ with $\boldsymbol{\beta }_{Q}$, $s_{M}$ with $s_{M_0}$, $s_{Q^\mathrm{F}}$ with $s_{Q}$, and $s_{M Q^\mathrm{F}}$ with $s_{M_0 Q}$. The covariance using the IMP method can be estimated (see Web Appendix A.2.2 for the proof) by $\widehat{\mathrm{Cov}}(\hat{\boldsymbol{\beta }}^{\mathrm{IMP}}_{M_{0}},\hat{\boldsymbol{\beta }}^{\mathrm{IMP}}_{Q})=\frac{1}{n} (\hat{I}^{M_0}_{0})^{-1} \hat{I}_1^{M_{0}Q} (\hat{I}^{Q}_{0})^{-1}$, where $\hat{I}^{M_0}_{0}=\frac{1}{n}\sum _{i=1}^n {\boldsymbol{Z}^{M_0}_{i}}^{\otimes 2}$, $\hat{I}^Q_{0}=\frac{1}{n}\sum _{i=1}^n {\boldsymbol{Z}^{Q}_{i}}^{\otimes 2}$,


(9) \begin{eqnarray*}
\hat{I}_1^{M_0 Q}&= & \frac{1}{n} \sum_{i=1}^n \frac{\Delta_{0 i} D_i\left(\hat{\boldsymbol{\beta}}_Q^{\mathrm{IMP}}\right) \otimes D_i\left(\hat{\boldsymbol{\beta}}_{M_0}^{\mathrm{IMP}}\right)}{\widehat{K}_0\left(T_i\right)}\\&&-\frac{1}{n^2} \sum_{i=1}^n \frac{\Delta_i D_i\left(\hat{\boldsymbol{\beta}}_Q^{\mathrm{IMP}}\right)}{\widehat{K}\left(T_i\right)} \otimes \sum_{i=1}^n \frac{\Delta_{0 i} D_i\left(\hat{\boldsymbol{\beta}}_{M_0}^{\mathrm{IMP}}\right)}{\widehat{K}_0\left(T_i\right)} \\&& +\widehat{J}^{C_0}\left\{D\left(\hat{\boldsymbol{\beta}}_Q^{\mathrm{IMP}}\right) \otimes D\left(\hat{\boldsymbol{\beta}}_{M_0}^{\mathrm{IMP}}\right)\right\}\\&&-\widehat{J}^{C_0}\left\{D\left(\hat{\boldsymbol{\beta}}_Q^{\mathrm{IMP}}\right) \otimes D\left(\hat{\boldsymbol{\beta}}_{M_0}^{\mathrm{IMP}}, u\right)\right\} \\&& -\widehat{J}^{C_0}\left\{D\left(\hat{\boldsymbol{\beta}}_Q^{\mathrm{IMP}}, u\right) \otimes D\left(\hat{\boldsymbol{\beta}}_{M_0}^{\mathrm{IMP}}\right)\right\}\\&&+\widehat{J}^{C_0}\left\{D\left(\hat{\boldsymbol{\beta}}_Q^{\mathrm{IMP}}, u\right) \otimes D\left(\hat{\boldsymbol{\beta}}_{M_0}^{\mathrm{IMP}}, u\right)\right\},
\end{eqnarray*}

\begin{eqnarray*}
\widehat{J}^{C_0}(X \otimes Y)&=&\frac{1}{n} \int _0^L\frac{dN^{CC_0}(u)}{\widehat{K}(u)^2}\left\lbrace \widehat{G}_0(X \otimes Y, u)\right.\\&&\left.-\widehat{G}(X, u) \otimes \widehat{G}_0(Y, u)\right\rbrace \frac{Y(u)}{Y_2(u)},
\end{eqnarray*}

$\widehat{G}_0(W,u)=\frac{1}{n\widehat{S}_0(u)}\sum _{i=1}^n \frac{\Delta _{0i}}{\widehat{K}_0(T_i)}W_i I(T_i\geqslant u)$ , $N^{CC_{0}}(u)=\sum _{i=1}^n N_i^{CC_{0}}(u)=\sum _{i=1}^n I(X_{0i}=X_{i}\leqslant u,\Delta _{0i}=\Delta _{i}=0)$, $Y_2(u)=\sum _{i=1}^n Y_{2i}(u)=\sum _{i=1}^n I(X_{i}\geqslant u, X_{0i}\geqslant u)$, and $\widehat{S}_0(u)$ is the KM estimator for survival function of $T_i$ at time *u*, $S_0(u)=\mathrm{Pr}(T_i> u)$, using data ($X_{0i}, \Delta _{0i}, i=1,\ldots , n$). Similar to $\widehat{J}$, if $\widehat{J}_0$ involves $D(\hat{\boldsymbol{\beta }}^{\mathrm{IMP}}_{V},u)$, $\widehat{G}_0$ is replaced by $\widehat{G}_0^{\star }(W,u)=\sum _{i=1}^n W_i Y_{0i}(u)/Y_{0}(u)$. For the SW method or using YOL as the effectiveness measure, the ICER and its statistical inference follow a similar manner as described in Section 2.3.3 (see Web Appendix A.2.2 for the proof).

### 2.5 INB and corresponding CI

Denoting $\lambda$ as the WTP for a unit gain of effectiveness (e.g. 1 year gain in event-free QAL), the covariate-adjusted $\mathrm{INB}(\lambda )$ can be estimated by $b_\lambda =\lambda \hat{\Delta }_{Q^\mathrm{F}}-\hat{\Delta }_M$, with a variance of $s^2_\lambda =\lambda ^2 s_{Q^\mathrm{F}}+s_{M}-2\lambda s_{M Q^\mathrm{F}}$. The Wald-type $100(1-2\alpha )\%$ CI of $\mathrm{INB}(\lambda )$ is given by $b_\lambda \pm z_\alpha s_\lambda$. The CIs of INB using event-free YOL as the effectiveness measure or under the scenario of different censoring times can be obtained similarly.

### 2.6 Subgroup identification with heterogeneous CE

Here, we discuss subgroup analysis for data with different terminating events, using event-free QAL as the effectiveness measure; similar approaches apply to other settings. When treatment-covariate interactions are present in model (1), CE becomes heterogeneous across patient subgroups, depending on covariates involved in the interaction. In this case, the subgroup-specific ICER for a patient subgroup with covariates $\boldsymbol{U}$ is


\begin{eqnarray*}
\gamma _{\boldsymbol{U}}=\frac{\Delta _M(\boldsymbol{U})}{\Delta _{Q^\mathrm{F}}(\boldsymbol{U})}=\frac{\beta _{M,A}+\boldsymbol{\beta }^{^{\prime }}_{M,A\boldsymbol{U}}\boldsymbol{U}}{\beta _{Q^\mathrm{F},A}+\boldsymbol{\beta }^{^{\prime }}_{Q^\mathrm{F},A\boldsymbol{U}}\boldsymbol{U}},
\end{eqnarray*}

where $\beta _{V,A}$ is the regression coefficient for the treatment indicator, and $\boldsymbol{\beta }^{^{\prime }}_{V,A\boldsymbol{U}}$ is the regression coefficient for interactions, which is 0 if no interactions included. Therefore, significant interactions can identify subgroups with heterogeneous CE, enabling the estimation of subgroup-specific ICERs by plugging in the estimated regression coefficients. Similarly, subgroup-specific INBs can be obtained. The statistical inferences for subgroup-specific ICERs and INBs rely on the estimated variances of $\hat{\Delta }_M(\boldsymbol{U})$, $\hat{\Delta }_{Q^\mathrm{F}}(\boldsymbol{U})$, and their covariance. These estimates can be obtained directly from $\widehat{\mathrm{Var}}(\hat{\boldsymbol{\beta }}_{M})$, $\widehat{\mathrm{Var}}(\hat{\boldsymbol{\beta }}_{Q^\mathrm{F}})$, and $\widehat{\mathrm{Cov}}(\hat{\boldsymbol{\beta }}_{M},\hat{\boldsymbol{\beta }}_{Q^\mathrm{F}})$, as $\hat{\Delta }_M(\boldsymbol{U})$ and $\hat{\Delta }_{Q^\mathrm{F}}(\boldsymbol{U})$ are linear combinations of regression coefficient estimators.

The proposed method is particularly advantageous for addressing confounding or characterizing heterogeneous CE by covariate adjustment. When no covariate adjustment is needed, the model simplifies to an unadjusted regression with an intercept and treatment indicator. A commonly used unadjusted alternative for CEA is treatment-stratified estimation of mean costs and effectiveness (Wang and Zhao, 2006; Chen and Zhao 2013). For a single categorical covariate *U*, unadjusted subgroup analyses within each level of *U* yield consistent estimates if no other confounders exist. However, this approach fails with multiple or continuous covariates, as it cannot address remaining confounding and heterogeneous effects. In contrast, our regression framework allows simultaneous adjustment for multiple covariates and flexible subgroup identification, providing more efficient and generalizable CE estimation.

## 3 Simulation

We conduct simulation studies to evaluate the performance of our proposed methods, based on 2000 replications with different sample sizes (*n* = 400, 800, 1200). The overall survival (OS) time until death has an exponential distribution with the rate parameter of $1/\mathrm{exp}(2.2-0.3A-0.5U+1.2A\times U)$. Treatment *A* and patient characteristic *U* are generated independently from a Bernoulli(0.5) distribution. Survival time is then truncated at $L=10$. The entire time period [0,10] is partitioned into 10 equal yearly intervals. For each interval, the patient’s utility weight $q_i(t)$ used to obtain QAL comprises a fixed weight (0.9 for $A=1$, 0.6 for $A=0$) and a random weight following $\mathrm{Uniform}(0, 0.1)$. U-shaped sample paths are adopted for the cost distribution (Chen and Zhao, 2013). The total costs of each patient consist of initial diagnostic costs at time 0, fixed patient-specific annual costs, random annual costs, and terminal costs incurred in the final year of life. To reflect higher initial costs for the new treatment, diagnostic costs are log-normally distributed with parameters $(8.5, 0.2^2)$ for $A=1$ and $(7.5, 0.2^2)$ for $A=0$. Random annual and terminal costs for all patients follow $\mathrm{log-normal}(4,0.2^2)$ and $\mathrm{log-normal}(7.2,0.4^2)$ distributions. The fixed annual costs are log-normally distributed with parameters $(6.8, 0.2^2)$, $(6.5, 0.2^2)$, $(7.2, 0.2^2)$, and $(6, 0.2^2)$ for the $\lbrace U=0, A=0\rbrace$, $\lbrace U=1, A=0\rbrace$, $\lbrace U=0, A=1\rbrace$, and $\lbrace U=1, A=1\rbrace$ groups, respectively.

### 3.1 Different terminating events

For illustrative purposes, consider heart failure (HF) as the event occurring before death. The time to the potential HF event is generated from an exponential distribution with the rate parameter of $1/\mathrm{exp}\lbrace 0.8\times (2.2-0.5U)+3\times (-0.3A+1.2A\times U)\rbrace$, truncated at $L=10$. In this setting, treatment has a greater influence on time to the HF event, with greater heterogeneity across patient subgroups compared to its effect on OS. We evaluate two censoring cases. The heavy censoring case, $C \sim \mathrm{Uniform}(0,13)$, yields approximately 44.1% censoring for OS and costs, and 26.8% for HF-free survival. In the light censoring case, $C \sim \mathrm{Uniform}(0,24)$, these rates were approximately 23.7% and 14.3%, respectively. True mean costs and effectiveness for all combinations of treatment *A* and characteristic *U* are presented in Web Appendix B (with the mathematical derivation in Web Appendix E). We then obtain the true coefficients $\boldsymbol{\beta }_{V}$ for costs, HF-free survival time, and HF-free QAL to derive the true ICERs.

To evaluate the performance of our proposed covariance estimators, we compare the sample covariance (SCV) between the mean cost and mean effectiveness estimators with the average estimated covariance (ECV) obtained from 2000 simulations. We also construct 95% CIs for the ICER as described in Section 2.3.3 in each simulation and compute the coverage probability (CP) of the true ICER. Since the ICER is a ratio statistic with a highly skewed distribution, we evaluate its accuracy using median bias, defined as the difference between the true ICER and the median of the estimated ICERs from 2000 simulations based on their positions on the CE plane. Efficiency is evaluated via the median CI wedge angle. Table 1 summarizes HF-free QAL results for our proposed methods, subgroup analysis, and unadjusted method. Our model incorporates treatment, covariate, and their interaction term. As expected, all SCVs are close to the ECVs, and the CPs are close to the nominal level of 95%, demonstrating the good performance of our proposed methods. Under heavy censoring, the IMP method yields CPs closer to nominal level and narrower CIs than the SW and subgroup methods. With light censoring and $n=400$, IMP slightly improves CPs. However, at larger sample sizes ($n=800$ and 1200), the SW method already achieves satisfactory CPs under light censoring, with the IMP method offering little improvement. Note that in the $U=0$ subgroup, the treatment is more costly and less effective, representing a dominated intervention on the CE plane. Because this treatment is clinically and economically inferior regardless of the WTP threshold, the ICER and its CI are not decision-relevant; these results are included to illustrate the proposed method’s finite-sample performance under dominance. Because the data-generating mechanism involves a treatment-covariate interaction, the unadjusted method ignoring *U* yields biased subgroup-specific ICERs and low 95% CI coverage. The subgroup approach analyzes the $U=0$ and $U=1$ groups separately using a simple weighted method without covariate adjustment. It performs similarly to our SW regression method in this setting with a single binary covariate. In Web Appendix B.1, we further examine a more complex simulation scenario involving an additional confounder. Our proposed methods achieve lower bias and higher CPs than the subgroup approach, which performs worse due to its inability to adjust for the remaining confounder.

**Table 1:** Summary of simulation results for ICER estimation, including median bias, covariance between costs and HF-free QAL, empirical coverage probabilities of 95% CIs, and median CI angle under different terminating events from 2000 simulations.

			Light censoring					Heavy censoring				
*n*	*U*	Method	Bias	SCV	ECV	CP	Angle	Bias	SCV	ECV	CP	Angle
400	0	Unadjusted	11.251	5	6	0.000	0.321	11.233	23	18	0.001	0.432
		Subgroup	−0.027	65	70	0.945	0.261	0.453	110	97	0.935	0.327
		SW	−0.074	69	73	0.942	0.269	–0.128	111	113	0.926	0.355
		IMP	−0.189	89	89	0.947	0.269	–0.173	130	128	0.942	0.319
	1	Unadjusted	1.215	5	6	0.001	0.321	1.197	23	18	0.019	0.432
		Subgroup	−0.002	52	58	0.941	0.207	0.004	86	84	0.939	0.276
		SW	−0.001	59	59	0.939	0.206	0.005	87	87	0.938	0.279
		IMP	−0.001	93	92	0.946	0.218	0.004	151	153	0.941	0.272
800	0	Unadjusted	11.228	3	3	0.000	0.227	11.229	10	9	0.000	0.305
		Subgroup	−0.033	37	36	0.945	0.183	0.197	55	52	0.940	0.226
		SW	−0.029	39	37	0.946	0.187	0.089	66	59	0.938	0.246
		IMP	0.010	46	44	0.946	0.185	0.027	68	65	0.949	0.216
	1	Unadjusted	1.192	3	3	0.000	0.227	1.193	10	9	0.000	0.305
		Subgroup	−0.000	29	29	0.951	0.145	0.000	40	41	0.945	0.199
		SW	−0.000	29	29	0.951	0.146	0.003	41	43	0.941	0.200
		IMP	0.002	46	46	0.959	0.153	0.002	75	75	0.949	0.193
1200	0	Unadjusted	11.235	0	2	0.000	0.184	11.228	4	6	0.000	0.246
		Subgroup	−0.085	23	24	0.951	0.148	0.134	35	35	0.941	0.183
		SW	−0.141	22	25	0.947	0.151	−0.137	41	39	0.944	0.197
		IMP	−0.130	29	30	0.949	0.149	−0.111	45	43	0.940	0.172
	1	Unadjusted	1.199	0	2	0.000	0.184	1.192	4	6	0.000	0.246
		Subgroup	0.001	20	20	0.949	0.118	0.002	28	28	0.949	0.160
		SW	−0.000	20	20	0.949	0.119	0.001	31	29	0.952	0.163
		IMP	0.002	31	31	0.954	0.125	0.002	51	50	0.950	0.157

*U* denotes the subgroup; Bias is the difference between the true ICER (−9.47 for $U=0$ and 0.55 for $U=1$, in $1000/year) and the median of estimates from 2000 simulations; SCV is the sample covariance between the mean cost estimators and the mean HF-free QAL estimators from 2000 simulations; ECV is the average of the estimated covariances obtained by our method; CP is the proportion containing the true ICER within the 95% CI; Angle is the median wedge angle of the CIs; SW denotes the simple weighted estimator for both costs and HF-free QAL; IMP refers to the improved estimator for both costs and HF-free QAL; Subgroup represents the separate simple weighted analysis within $U=0$ and $U=1$; Unadjusted represents simple weighted analysis without adjustment for covariate *U*.

The bias for estimates and standard errors (SEs) of the regression coefficients used in deriving the ICER and its CI are also provided and compared in the Web Appendix D.1. As noted by Wang and Zhao (2007), the IMP estimator does not guarantee improved efficiency in all cases. In our simulations, the IMP estimator outperforms the SW estimator on regression coefficients for HF-free QAL, especially under heavy censoring. For cost, the IMP method exhibits smaller bias and improved CPs in small samples with heavy censoring, but does not show superiority to the SW estimators in larger samples or scenarios with light censoring. The simulation results using HF-free YOL as the effectiveness measure for different terminating events are provided in Web Appendix B.1 with similar conclusions.

### 3.2 Different censoring times

We generate the cost censoring time as $C_0={\rm min}(C, \tilde{C}_0)$, where the potential early-censoring time $\tilde{C}_0 \sim \mathrm{Uniform}(0,15)$ and the censoring time for survival $C \sim \mathrm{Uniform}(0,30)$. If $\tilde{C}_0> C$, then $C_0=C$, meaning that costs are collected until the last survival follow-up. The generation process of costs, survival time, and QAL is the same as in Section 3.1, leading to about 43.5% and 19.0% censoring in early-censored costs and survival time, respectively.

Table 2 presents the results using QAL and YOL as the effectiveness measures. The ECVs are close to the SCVs. For our proposed method, the IMP estimator yields slightly better 95% CPs than the SW estimator but produces wider median CI wedge angles, potentially explaining the marginal CP increase. The unadjusted approach ignores *U* results in large median bias and poor CPs for subgroup-specific ICERs. Under this simulation setting with one binary covariate, subgroup analysis performs similarly to our SW method. Web Appendix B.2 examines a setting with an additional confounder, where our methods outperform subgroup analysis when further adjustment is needed. Our methods are more efficient than the commonly used naive approach of censoring survival at the cost censoring time. As shown in Web Appendix B.2, the naive method results in an efficiency loss (measured by SE) of 46.9%–51.3% for mean QAL and 52.8%–57.4% for mean YOL estimation.

**Table 2:** Summary of simulation results for ICER estimation, including median bias, covariance between early-censored costs and effectiveness, empirical coverage probabilities of 95% CIs, and median CI angle under different censoring time from 2000 simulations.

			QAL					YOL				
*n*	*U*	Method	Bias	SCV	ECV	CP	Angle	Bias	SCV	ECV	CP	Angle
400	0	Unadjusted	−3.227	84	86	0.001	0.328	10.309	109	110	0.122	0.394
		Subgroup	−0.013	291	281	0.943	0.321	−0.128	355	339	0.944	0.577
		SW	−0.069	282	280	0.944	0.319	−0.125	343	337	0.943	0.574
		IMP	−0.035	326	331	0.949	0.331	−0.248	348	345	0.951	0.590
	1	Unadjusted	0.892	84	86	0.064	0.328	3.608	109	110	0.003	0.394
		Subgroup	0.003	103	102	0.943	0.307	0.006	134	136	0.943	0.420
		SW	0.001	100	102	0.945	0.308	0.004	133	136	0.942	0.418
		IMP	−0.003	138	141	0.954	0.347	−0.005	140	145	0.947	0.463
800	0	Unadjusted	−3.248	41	43	0.000	0.229	10.211	51	55	0.005	0.270
		Subgroup	0.015	136	141	0.953	0.216	−0.102	164	170	0.955	0.395
		SW	0.055	136	141	0.953	0.215	−0.113	165	169	0.956	0.394
		IMP	0.049	164	165	0.953	0.221	−0.143	168	173	0.953	0.401
	1	Unadjusted	0.871	41	43	0.001	0.229	3.510	51	55	0.000	0.270
		Subgroup	0.001	52	51	0.953	0.215	0.000	69	68	0.958	0.286
		SW	−0.000	51	51	0.948	0.217	0.002	68	68	0.955	0.287
		IMP	−0.002	64	71	0.944	0.243	−0.003	67	73	0.944	0.315
1200	0	Unadjusted	−3.246	29	29	0.000	0.187	10.330	38	37	0.001	0.220
		Subgroup	−0.010	99	94	0.949	0.173	0.014	121	113	0.943	0.321
		SW	−0.018	101	94	0.950	0.173	0.007	123	113	0.941	0.321
		IMP	−0.019	115	110	0.949	0.177	−0.057	121	115	0.947	0.323
	1	Unadjusted	0.873	29	29	0.000	0.187	3.629	38	37	0.000	0.220
		Subgroup	0.001	33	34	0.946	0.175	0.001	44	46	0.946	0.232
		SW	0.000	34	34	0.942	0.176	0.003	45	45	0.945	0.231
		IMP	−0.001	47	47	0.951	0.197	−0.002	49	49	0.951	0.255

*U* denotes the subgroup; Bias is the difference between the true ICER and the median of estimates from 2000 simulations; the true ICER values are 4.83 and 0.71 (in $1000/year) for $U=0$ and $U=1$, respectively, when QAL is used as the effectiveness measure, and −5.62 and 1.07 (in $1000/year) for $U=0$ and $U=1$, respectively, when YOL is used as the effectiveness measure; SCV is the sample covariance between the mean early-censored cost estimators and the mean effectiveness (QAL and YOL) estimators from 2000 simulations; ECV is the average of the estimated covariances obtained by our method; CP is the proportion containing the true ICER within the 95% CI; Angle is the median wedge angle of the CIs; SW denotes the simple weighted estimator for early-censored costs, QAL, and YOL; IMP refers to the improved estimator for early-censored costs and QAL only; Subgroup represents the separate simple weighted analysis within $U=0$ and $U=1$; Unadjusted represents simple weighted analysis without adjustment for covariate *U*.

Web Appendix D.2 presents the bias for regression coefficient estimates and SEs used in deriving the ICER and its CI. For small samples, the IMP method yields higher CPs for costs and QAL than the SW method. With larger samples, the CPs of both methods approach the nominal level. The IMP method does not show improved efficiency. These results align with Table 2, where the IMP method provides better CPs but slightly wider CIs.

For both data scenarios with different terminating events and censoring times, we further compare our methods with two bootstrap methods at $n=400$. The results (Web Appendix B) show that the bootstrap percentile method performs poorly in subgroup $U=0$. This issue arises in ICER as the percentile method always yields finite CIs, which is misleading when the treatment effect is not significant (Fan and Zhou, 2007). This limitation does not affect INB. While the reordered bootstrap method (Wang and Zhao, 2008) yields comparable results, our methods run significantly faster, taking 6.3 min on a single core versus 7.4 h on 30 cores for the bootstrap ($\approx$222 h on a single core). All computations were performed in R 4.4.0 on a 32-core Linux server with 251 GiB RAM. We also assessed our methods’ performance in estimating INB (Web Appendix C) with similar findings.

## 4 Examples

### 4.1 MADIT-CRT with different terminating events

In the MADIT-CRT trial, patients were randomized to receive CRT with an implantable cardiac defibrillator (CRT–ICD) or ICD-only (ICD) in a 3:2 ratio. After trial completion, CRT–ICD was shown to reduce the risk of HF or death, particularly in patients with left bundle branch block (LBBB) (Zareba et al., 2011). Given the high cost of ICD implantation, a CEA was conducted with 748 patients in the CRT–ICD arm and 503 in the ICD arm from US centers (Noyes et al., 2013). In this cohort, 9% in the ICD–CRT arm and 22% in the ICD arm experienced HF but continued to accrue costs. The patient health-related quality of life was assessed using the EQ-5D instrument (Shaw et al., 2005). Cost data were collected with start and stop dates for each entry, assumed evenly distributed over each interval, while health utilities remained constant until the next assessment and dropped to zero at death. Costs, survival time, and QAL were discounted at a 3% annual rate.

We applied our methods to evaluate the CE of CRT–ICD versus ICD within a 4-year limit adjusting for LBBB status. The regression models included the treatment indicator, LBBB status, and their interaction to account for potential heterogeneity across LBBB status. Excluding 1 patient with missing status, the cohort included 859 LBBB and 391 non-LBBB patients. The censoring rate at the 4-year horizon was 84.7% for OS and costs, and 72.9% for HF-free survival. We used both SW and IMP methods to estimate the mean costs (Section 2.2) and HF-free QAL (Section 2.3), and applied the SW estimator (Section 2.3.1) to HF-free YOL. The results for LBBB and non-LBBB groups using HF-free QAL as the effectiveness measure are presented in Table 3. The IMP method yielded smaller SEs for cost estimates and comparable SEs for HF-free QAL compared to the SW method, suggesting improved efficiency in this heavily censored dataset. A significant treatment-by-LBBB interaction in the effectiveness model indicated heterogeneity in HF-free QAL. Specifically, CRT–ICD was associated with higher HF-free QAL in the LBBB group, whereas a nonsignificant reduction in HF-free QAL was observed in the non-LBBB group.

**Table 3:** Estimated mean total costs ($1000), HF-free QAL (year), ICERs ($1000/year), and CIs, limited to a 4-year time horizon, for LBBB and non-LBBB groups in the MADIT-CRT data with different terminating events.

		CRT–ICD		ICD		Difference	
		Estimate (SE)		Estimate (SE)		Estimate (SE)	
		SW	IMP	SW	IMP	SW	IMP
LBBB group							
Cost		55.5 (2.63)	62.3 (1.78)	56.5 (4.32)	59.6 (1.89)	−1.0 (5.06)	2.7 (2.67)
HF-free QAL		3.0 (0.03)	3.1 (0.03)	2.6 (0.04)	2.7 (0.04)	0.5 (0.15)	0.4 (0.15)
Non-LBBB group							
Cost		64.1 (3.07)	67.2 (2.79)	62.6 (8.48)	57.4 (3.45)	1.4 (9.02)	9.8 (4.50)
HF-free QAL		2.4 (0.05)	2.5 (0.04)	2.8 (0.06)	2.7 (0.06)	−0.4 (0.22)	−0.2 (0.22)
	LBBB group			Non-LBBB group			
Method	ECV	ICER (95% CI)		ECV	ICER (95% CI)		
Subgroup	−0.242	−2.2 (−20.9, 26.7)		−1.011	−2.3 $(-\infty , +\infty )$		
SW	−0.270	−2.1 $(-23.1, 31.7)$		−0.730	−3.7 $(-\infty , +\infty )$		
IMP	0.003	7.0 $(-8.1, 43.9)$		0.221	−50.1 $(-\infty , -3.0)\cup (41.6, +\infty )$		

SE is the estimated standard error; ECV is the estimated covariance between cost and effectiveness; SW denotes the simple weighted estimator; IMP refers to the improved estimator; Our methods include treatment, LBBB status, and their interaction in the model; Subgroup refers to subgroup simple weighted analysis within LBBB and non-LBBB groups.

In the LBBB group, CRT–ICD was more effective but more costly than ICD. The IMP method yielded an estimated ICER of $6981/year, with the 95% CI spanning the first and fourth quadrants of the CE plane, indicating potential CE depending on the WTP threshold. The SW method produced a negative cost difference, placing the point estimate of ICER (−$2113/year) in the fourth quadrant. Compared with the IMP method, the SW method was less efficient, yielding a wider 95% CI wedge angle (SW: 3.067 vs. IMP: 2.996). In the non-LBBB group, the treatment was dominated, being more costly and less effective, rendering the ICER and its CI uninformative for decision-making. While CEAs are often omitted under dominance, we include these results for illustration purposes of the proposed methods. In this group, the mean difference in HF-free QAL was close to zero, leading to a negative denominator in Equation (7). As the corresponding discriminant was positive for the IMP method, Fieller’s method yielded a disjoint confidence set $(-\infty , -\$2{,}988)\cup (\$41{,}620, +\infty )$ per year. Geometrically, this occurs when the joint confidence region for incremental cost and effectiveness includes the vertical axis of the CE plane, implying an unbounded CI. For the SW method, a larger estimated SE of the mean cost difference produced a negative discriminant, yielding an uninformative CI that spans the entire real line. In addition, we conducted subgroup analyses using the unadjusted simple weighted method within the LBBB and non-LBBB groups, yielding results comparable to those from our SW regression method.

**Figure 1:**
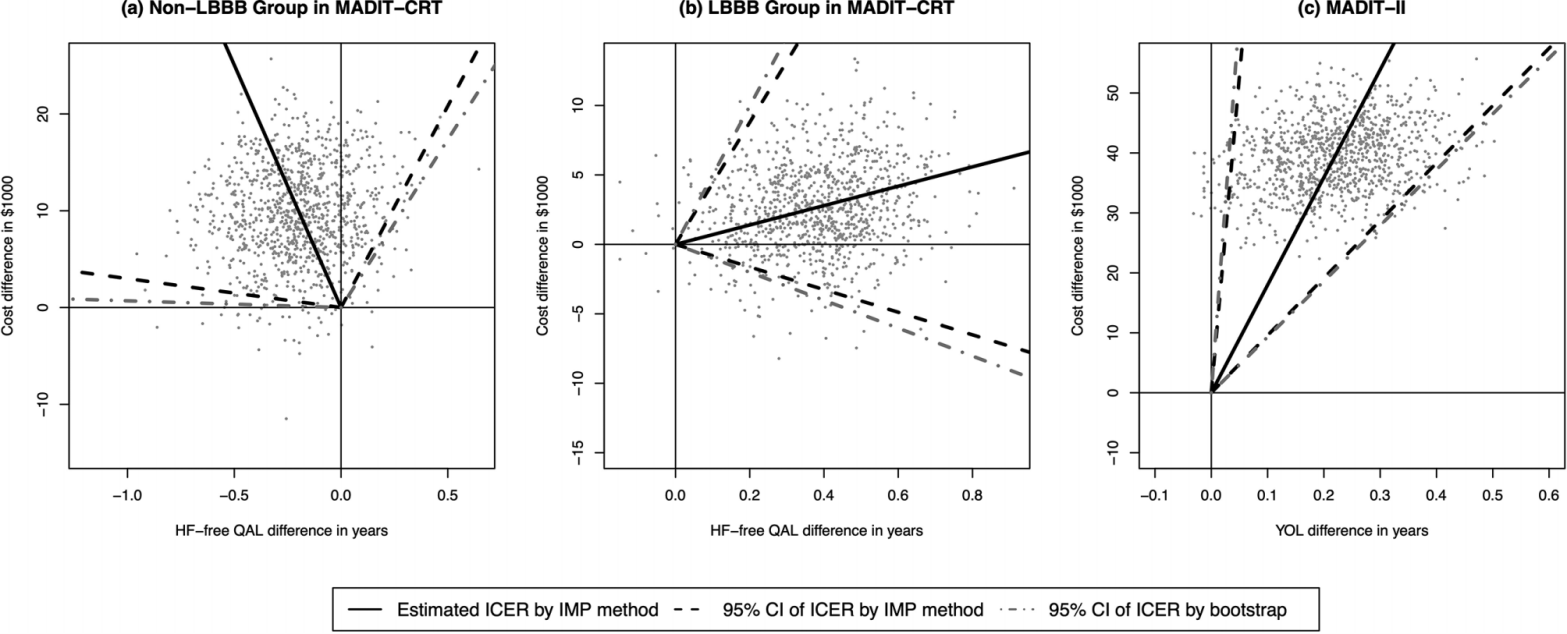
Estimated ICERs and 95% CIs for the MADIT-CRT and MADIT-II trials. The dots represent 1000 bootstrap samples. The solid lines denote the estimated ICERs; the black dashed lines and the gray dot-dashed lines indicate CI limits obtained by the IMP method and the bootstrap method, respectively. Panels (A) and (B) present results using HF-free QAL as the effectiveness measure for the LBBB and non-LBBB subgroups in the MADIT-CRT study (4-year time horizon). Panel (C) presents results using YOL as the effectiveness measure in the MADIT-II study (3.5-year time horizon).

We additionally applied the angle-based bootstrap method (Fan and Zhou, 2007) to obtain the 95% CIs of ICERs. This approach is useful when bootstrap samples span all quadrants of the CE plane, as observed in this example. Figure 1(A) and 1(B) presents the bootstrap samples from the IMP method, along with ICER estimates and 95% CIs from both the IMP and Bootstrap methods using HF-free QAL. The CIs from our method closely matched but were narrower than the bootstrap CIs (LBBB: $(-\$10,054, \$49,796)$; non-LBBB: $(-\infty , -\$692) \cup (\$34,706, +\infty )$). The results using HF-free YOL as the effectiveness measure are presented in Web Appendix B.1 with similar findings. Web Appendix B.1 also includes results evaluating unrestricted YOL and QAL, where the mean effectiveness differences within the LBBB subgroup were no longer significant, consistent with previous findings that CRT–ICD reduced HF but did not significantly extend OS.

### 4.2 MADIT-II with different censoring times

In MADIT-II, patients were randomized to either ICD or conventional therapy. Moss et al. (2002) demonstrated significant survival benefits for ICD over the conventional group, followed by a CEA of US-based patients (Zwanziger et al., 2006). The censoring rates were 73.9% for survival and 75.7% for costs. A 3% annual discount rate was applied to both survival time and costs. We applied the SW and IMP estimators for early-censored costs (Section 2.4) and the SW estimator for YOL (Section 2.2) to estimate ICER, with a maximum follow-up of 3.5 years. The analysis adjusted for several binary baseline factors, including gender, HF status, blood urea nitrogen levels, and ejection fraction. After excluding subjects with missing covariates, the cohort comprised 655 ICD and 420 conventional therapy patients.

Table 4 shows that the ICD arm incurred significantly higher costs and longer YOL than the conventional arm, indicating a trade-off between cost and effectiveness. The estimated covariate-adjusted ICERs were $145 300/year and $179 600/year using the SW and IMP methods, respectively, suggesting that ICD would be considered cost-effective only at relatively high WTP thresholds. The IMP method demonstrated reduced uncertainty, as reflected by a narrower 95% CI wedge angle for the ICER (SW: 0.015 vs. IMP: 0.009). Additionally, the IMP CI closely aligned with the 95% reordered bootstrap CI ($93 040, $1 261 580), as shown in Figure 1(C). This example focuses on covariate-adjusted CE rather than subgroup-specific estimation. We therefore also evaluated two naive approaches: an unadjusted analysis that ignores covariates and an approach that censors survival at the cost-censoring time. Both naive methods produced larger SEs for YOL differences. The lack of covariate adjustment led to nonsignificant YOL differences with unbounded ICER CI. These results highlight the efficiency gains from covariate adjustment in our methods. The results for INB are provided in Web Appendix C with similar conclusions.

**Table 4:** Estimated mean difference (ICD vs. conventional treatment) in early-censored costs ($1000) and YOL (year), ICERs ($1000/year), and CIs, limited to a 3.5-year time horizon, for the MADIT-II data with different censoring times.

		Difference estimate (SE)			
Method		Cost	YOL	ECV	ICER (95% CI)
Our method	SW	31.9 (6.9)	0.220 (0.094)	0.002	145.3 (63.6, 922.2)
	IMP	39.4 (5.6)	$-$	0.126	179.6 (95.8, 1056.4)
Early censor	SW	31.9 (6.9)	0.212 (0.096)	0.002	150.3 (64.4, 1349.6)
	IMP	39.4 (5.6)	$-$	0.131	185.8 (97.0, 1547.6)
Unadjusted		33.1 (7.4)	0.174 (0.097)	−0.068	189.6 $(-\infty , -2168.6) \cup (70.7, +\infty )$

Difference estimate is the estimated (covariate-adjusted) mean difference between ICD and Conventional; SE is estimated standard error; ECV is the estimated covariance between cost and effectiveness; SW denotes the simple weighted estimator; IMP refers to the improved estimator; Our methods include treatment, gender, heart failure, blood urea nitrogen, and ejection fraction in the model; Early-censor method refers to censoring the survival at the cost-censoring time; Unadjusted refers to simple weighted analysis without adjustment for covariates.

## 5 Discussion

Different terminating events for effectiveness and costs are common in both randomized and observational studies. Beyond our cardiovascular study examples, similar scenarios arise in cancer, transplant, and neurological research. Early censored cost is also frequently observed, and naive approaches that censor survival early can reduce statistical efficiency and misinform healthcare decisions. To address these challenges, we proposed regression methods for CEA of censored data from randomized or observational studies with different terminating events or censoring times, where effectiveness is measured by survival time or QAL. These methods construct accurate CIs for the ICER and INB while allowing covariate adjustment and subgroup identification under heterogeneous CE. As a result, they improve the estimation efficiency and provide reliable coverages for the CIs of ICER and INB, as demonstrated in simulations. The SW estimator is useful and convenient when only the total costs or QAL are available, whereas the IMP estimator is generally more efficient when cost or QAL history is observed, particularly under high censoring or small sample sizes.

The proposed methods are comparable to commonly used bootstrap approaches in performance, while improving computational efficiency and avoiding the error-prone sample rearrangement on the CE plane required by bootstrap methods to obtain correct CIs. In the MADIT-CRT trial with different terminating events, our methods adjust for covariates to improve estimation efficiency, identify the LBBB subgroup as particularly cost-effective, and avoid computationally intensive bootstrap resampling for CI construction. In the MADIT-II trial with different censoring times, our methods efficiently adjust for several baseline covariates and outperform the naive approach that censors survival time early. In contrast, traditional unadjusted methods like simple subgroup analyses cannot handle multiple or continuous covariates. Overall, the proposed methods offer valuable analytical tools for CEA in complex censored data with different terminating events or censoring times from randomized or observational studies, leveraging additional information to produce more accurate and efficient economic evaluations that support evidence-based decisions and resource allocation in clinical and policy contexts.

A few extensions of this work are possible. Linear models may be inappropriate in some settings, and our approach could integrate generalized linear models for cost data (Lin, 2003) or other nonlinear models to estimate longitudinal cost trajectories (Li et al., 2018). Additionally, applying doubly robust methods to assess average causal CE can improve the validity of statistical inferences in observational studies (Chen and Hoch, 2022).

## Supplementary Material

ujag073_Supplemental_Files Sample R codes for implementing the proposed method and Web Appendices referenced in Sections 2–4 are available with this paper at the Biometrics website on Oxford Academic.

## Data Availability

While the data transfer and use agreements prohibit making the MADIT-CRT and MADIT-II datasets publicly available, Boston Scientific may share the data with qualified researchers, available at https://www.bostonscientific.com/en-US/data-sharing-requests.html
