# Supplementary material for: Regression methods for cost-effectiveness analysis with different censoring times or terminating events for survival time and costs
Source: Biometrics. 2026 May 12;82(2):ujag073. doi: 10.1093/biomtc/ujag073 (PMC13164937; doi:10.1093/biomtc/ujag073)
Supplement: ujag073_Supplemental_Files — Sample R codes for implementing the proposed method and Web Appendices referenced in Sections 2–4 are available with this paper at the Biometrics website on Oxford Academic. [file ujag073_supplemental_files.zip › WebSupp.pdf]

Supplementary Materials for "Regression methods for cost-effectiveness analysis with different censoring times or terminating events for survival time and costs" by

Dingning Liu and Shuai Chen

Web Appendix A: Estimation of the variance and covariance matrix of effectiveness and costs

A.1.1: Variance estimators for data with different terminating events

The explicit formula for the IMP estimator of the regression coefficients for event-free QAL (Q^{F}) from Section 2.3.2 of the main text is $\hat{\boldsymbol{\beta}}^{\mathrm{IMP}}_{Q^{\mathrm{F}}} = (\mathbf{B}_1^{Q^{\mathrm{F}}})^{-1}\mathbf{B}_2^{Q^{\mathrm{F}}}$, where

$$\mathbf{B}_1^{Q^{\mathrm{F}}} = \sum_{i=1}^{n}\left\{\frac{\Delta_i^{\mathrm{F}}}{\widehat{K}^{\mathrm{F}}(T_i^{\mathrm{F}})} + \int_0^L \frac{\mathrm{d}N_i^{\mathrm{F}}(u)}{\widehat{K}^{\mathrm{F}}(u)}\right\}\boldsymbol{Z}_i^{Q^{\mathrm{F}}\otimes 2} - \sum_{i=1}^{n}\int_0^L \frac{\sum_{j=1}^{n} Y_j^{\mathrm{F}}(u)\boldsymbol{Z}_j^{Q^{\mathrm{F}}\otimes 2}}{\widehat{K}^{\mathrm{F}}(u)Y^{\mathrm{F}}(u)}\mathrm{d}N_i^{\mathrm{F}}(u),$$

$$\mathbf{B}_2^{Q^{\mathrm{F}}} = \sum_{i=1}^{n}\left\{\frac{\Delta_i^{\mathrm{F}}Q_i^{\mathrm{F}}}{\widehat{K}^{\mathrm{F}}(T_i^{\mathrm{F}})} + \int_0^L \frac{Q_i^{\mathrm{F}}(u)}{\widehat{K}^{\mathrm{F}}(u)}\mathrm{d}N_i^{\mathrm{F}}(u)\right\}\boldsymbol{Z}_i^{Q^{\mathrm{F}}} - \sum_{i=1}^{n}\int_0^L \frac{\sum_{j=1}^{n} Q_j^{\mathrm{F}}(u)Y_j^{\mathrm{F}}(u)\boldsymbol{Z}_j^{Q^{\mathrm{F}}}}{\widehat{K}^{\mathrm{F}}(u)Y^{\mathrm{F}}(u)}\mathrm{d}N_i^{\mathrm{F}}(u),$$

and event-free at-risk process is defined as $Y^{\mathrm{F}}(u) = \sum_{i=1}^{n} Y_i^{\mathrm{F}}(u) = \sum_{i=1}^{n} I(X_i^{\mathrm{F}} \geq u)$. Following Lin (2000), the asymptotic variance-covariance matrix of $\hat{\boldsymbol{\beta}}^{\mathrm{SW}}_{Q^{\mathrm{F}}}$ can be estimated by

$\widehat{\text{Var}}(\hat{\boldsymbol{\beta}}_{Q^{\text{F}}}^{\text{SW}}) = \frac{1}{n}(\hat{I}_0^{Q^{\text{F}}})^{-1}\hat{I}_1^{Q^{\text{F}}}(\hat{I}_0^{Q^{\text{F}}})^{-1}$, where $\hat{I}_0^{Q^{\text{F}}} = \frac{1}{n}\sum_{i=1}^{n}\boldsymbol{Z}_i^{Q^{\text{F}}\otimes 2}$,

$$\hat{I}_1^{Q^{\text{F}}} = \frac{1}{n}\sum_{i=1}^{n}\frac{\Delta_i^{\text{F}}}{\widehat{K}^{\text{F}}(T_i^{\text{F}})}\{D_i(\hat{\boldsymbol{\beta}}_{Q^{\text{F}}}^{\text{SW}})\}^{\otimes 2} + \widehat{J}^{\text{F}}\{D(\hat{\boldsymbol{\beta}}_{Q^{\text{F}}}^{\text{SW}})\otimes D(\hat{\boldsymbol{\beta}}_{Q^{\text{F}}}^{\text{SW}})\},$$

$$\widehat{J}^{\text{F}}(X\otimes Y) = \frac{1}{n}\int_0^L\{\widehat{G}^{\text{F}}(X\otimes Y,u) - \widehat{G}^{\text{F}}(X,u)\otimes\widehat{G}^{\text{F}}(Y,u)\}\frac{\mathrm{d}N^{\text{F}}(u)}{\widehat{K}^{\text{F}}(u)^2},$$

$\widehat{G}^{\text{F}}(W,u) = \frac{1}{n}\frac{1}{\widehat{S}^{\text{F}}(u)}\sum_{i=1}^{n}\frac{\Delta_i^{\text{F}}W_iI(T_i^{\text{F}}\geq u)}{\widehat{K}^{\text{F}}(T_i^{\text{F}})}$, $N^{\text{F}}(u) = \sum_{i=1}^{n}N_i^{\text{F}}(u) = \sum_{i=1}^{n}I(X_i^{\text{F}}\leq u, \Delta_i^{\text{F}} = 0)$, and $\widehat{S}^{\text{F}}(u)$ is the KM estimator for the survival function of T_i^{F} at time u , $S^{\text{F}}(u) = \Pr(T_i^{\text{F}} > u)$, using data $(X_i^{\text{F}}, \Delta_i^{\text{F}}, i = 1,\ldots,n)$. The asymptotic variance-covariance estimator for event-free QAL $\hat{\boldsymbol{\beta}}_{Q^{\text{F}}}^{\text{IMP}}$ is $\widehat{\text{Var}}(\hat{\boldsymbol{\beta}}_{Q^{\text{F}}}^{\text{IMP}}) = \frac{1}{n}(\hat{I}_0^{Q^{\text{F}}})^{-1}\hat{I}_1^{Q^{\text{F}}}(\hat{I}_0^{Q^{\text{F}}})^{-1}$, where

$$\begin{aligned}\hat{I}_1^{Q^{\text{F}}} &= \frac{1}{n}\sum_{i=1}^{n}\frac{\Delta_i^{\text{F}}}{\widehat{K^{\text{F}}}(T_i^{\text{F}})}\{D_i(\hat{\boldsymbol{\beta}}_{Q^{\text{F}}}^{\text{IMP}})\}^{\otimes 2}\\ &\quad+\widehat{J}^{\text{F}}\{D(\hat{\boldsymbol{\beta}}_{Q^{\text{F}}}^{\text{IMP}})\otimes D(\hat{\boldsymbol{\beta}}_{Q^{\text{F}}}^{\text{IMP}})\} + \widehat{J}^{\text{F}}\{D(\hat{\boldsymbol{\beta}}_{Q^{\text{F}}}^{\text{IMP}},u)\otimes D(\hat{\boldsymbol{\beta}}_{Q^{\text{F}}}^{\text{IMP}},u)\}\\ &\quad-\widehat{J}^{\text{F}}\{D(\hat{\boldsymbol{\beta}}_{Q^{\text{F}}}^{\text{IMP}})\otimes D(\hat{\boldsymbol{\beta}}_{Q^{\text{F}}}^{\text{IMP}},u)\} - \widehat{J}^{\text{F}}\{D(\hat{\boldsymbol{\beta}}_{Q^{\text{F}}}^{\text{IMP}},u)\otimes D(\hat{\boldsymbol{\beta}}_{Q^{\text{F}}}^{\text{IMP}})\}.\end{aligned}$$

When $\widehat{J}^{\text{F}}$ involves $D(\hat{\boldsymbol{\beta}}_{Q^{\text{F}}}^{\text{IMP}},u)$, $\widehat{G}^{\text{F}}$ is replaced by $\widehat{G}^{\text{F}\star}(W,u) = \frac{\sum_{i=1}^{n}W_iY_i^{\text{F}}(u)}{Y^{\text{F}}(u)}$ to utilize health history from both censored and uncensored patients, while $\widehat{G}^{\text{F}}$ only includes uncensored patients (i.e., the summand in $\widehat{G}^{\text{F}}$ is 0 for censored patients with $\Delta_i^{\text{F}} = 0$).

A.1.2: Covariance estimators for data with different terminating events

The covariances of our proposed methods can be obtained following the idea of Bang and Tsiatis (2000) and Willan et al. (2005). Consider the martingale process $\mathcal{M}_i^C(u) = N_i^C(u) - \int_0^u\lambda^C(t)Y_i(t)\mathrm{d}t$ and $\mathcal{M}_i^{\text{F}}(u) = N_i^{\text{F}}(u) - \int_0^u\lambda^C(t)Y_i^{\text{F}}(t)\mathrm{d}t$, where the hazard function for the censoring distribution $\lambda^C(t) = \lim_{h\to 0}\frac{1}{h}\Pr(t < C < t+h|C\geq t, T\geq t)$. From Chen and Zhao (2013), $\text{Cov}\{d\mathcal{M}_i^C(u), d\mathcal{M}_i^{\text{F}}(u)\} = Y_i^{\text{F}}(u)\lambda^C(u)du$. To derive Equation (8) in the main

text using the IMP method for both costs and event-free QAL, we have

$$
\begin{aligned}
I_1^{MQ^{\mathrm{F}}} &= \mathrm{E}\{D_i(\boldsymbol{\beta}_{Q^{\mathrm{F}}}) \otimes D_i(\boldsymbol{\beta}_M)\} + \mathrm{E}\{W_1^{\mathrm{F}}(\boldsymbol{\beta}_{Q^{\mathrm{F}}}) \otimes W_1(\boldsymbol{\beta}_M)\} - \mathrm{E}\{W_1^{\mathrm{F}}(\boldsymbol{\beta}_{Q^{\mathrm{F}}}) \otimes W_2(\boldsymbol{\beta}_M)\} \\
&\quad - \mathrm{E}\{W_2^{\mathrm{F}}(\boldsymbol{\beta}_{Q^{\mathrm{F}}}) \otimes W_1(\boldsymbol{\beta}_M)\} + \mathrm{E}\{W_2^{\mathrm{F}}(\boldsymbol{\beta}_{Q^{\mathrm{F}}}) \otimes W_2(\boldsymbol{\beta}_M)\}, \qquad (1)
\end{aligned}
$$

where

$$
\begin{aligned}
W_1^{\mathrm{F}}(\boldsymbol{\beta}_{Q^{\mathrm{F}}}) &= \int_0^L [D_i(\boldsymbol{\beta}_{Q^{\mathrm{F}}}) - G^{\mathrm{F}}\{D(\boldsymbol{\beta}_{Q^{\mathrm{F}}}), u\}] \frac{\mathrm{d}\mathcal{M}_i^{\mathrm{F}}(u)}{K^{\mathrm{F}}(u)}, \\
W_2^{\mathrm{F}}(\boldsymbol{\beta}_{Q^{\mathrm{F}}}) &= \int_0^L [D_i(\boldsymbol{\beta}_{Q^{\mathrm{F}}}, u) - G^{\mathrm{F}}\{D(\boldsymbol{\beta}_{Q^{\mathrm{F}}}, u), u\}] \frac{\mathrm{d}\mathcal{M}_i^{\mathrm{F}}(u)}{K^{\mathrm{F}}(u)}, \\
W_1(\boldsymbol{\beta}_M) &= \int_0^L [D_i(\boldsymbol{\beta}_M) - G\{D(\boldsymbol{\beta}_M), u\}] \frac{\mathrm{d}\mathcal{M}_i^C(u)}{K(u)}, \\
W_2(\boldsymbol{\beta}_M) &= \int_0^L [D_i(\boldsymbol{\beta}_M, u) - G\{D(\boldsymbol{\beta}_M, u), u\}] \frac{\mathrm{d}\mathcal{M}_i^C(u)}{K(u)}.
\end{aligned}
$$

The second term of Equation (1) can be written as

$$
\begin{aligned}
&\mathrm{E}\int_0^L [D_i(\boldsymbol{\beta}_{Q^{\mathrm{F}}}) - G^{\mathrm{F}}\{D(\boldsymbol{\beta}_{Q^{\mathrm{F}}}), u\}][D_i(\boldsymbol{\beta}_M) - G\{D(\boldsymbol{\beta}_M), u\}] Y_i^F(u) \frac{\lambda^C(u)}{K^2(u)} du \\
&= \mathrm{E}\int_0^L [D_i(\boldsymbol{\beta}_{Q^{\mathrm{F}}}) D_i(\boldsymbol{\beta}_M) - G^{\mathrm{F}}\{D(\boldsymbol{\beta}_{Q^{\mathrm{F}}}), u\} D_i(\boldsymbol{\beta}_M) - D_i(\boldsymbol{\beta}_{Q^{\mathrm{F}}}) G\{D(\boldsymbol{\beta}_M), u\} \\
&\qquad + G^{\mathrm{F}}\{D(\boldsymbol{\beta}_{Q^{\mathrm{F}}}), u\} \otimes G\{D(\boldsymbol{\beta}_M), u\}] I(T_i^{\mathrm{F}} \geq u) \frac{\lambda^C(u)}{K(u)} du \\
&= \int_0^L [G^{\mathrm{F}}\{D(\boldsymbol{\beta}_{Q^{\mathrm{F}}}) \otimes D(\boldsymbol{\beta}_M), u\} - G^{\mathrm{F}}\{D(\boldsymbol{\beta}_{Q^{\mathrm{F}}}), u\} \otimes G^{\mathrm{F}}\{D(\boldsymbol{\beta}_M), u\}] S^{\mathrm{F}}(u) \frac{\lambda^C(u)}{K(u)} du,
\end{aligned}
$$

which can be estimated by $\widehat{J}^F\{D(\boldsymbol{\beta}_{Q^{\mathrm{F}}}) \otimes D(\boldsymbol{\beta}_M)\}$. The estimators for the last three terms of Equation (1) can be derived similarly. Therefore, the IMP estimator for $I_1^{MQ^{\mathrm{F}}}$ is given by

$$
\begin{aligned}
\hat{I}_1^{MQ^{\mathrm{F}}} &= \frac{1}{n}\sum_{i=1}^n \frac{\Delta_i D_i(\hat{\boldsymbol{\beta}}_{Q^{\mathrm{F}}}^{\mathrm{IMP}}) \otimes D_i(\hat{\boldsymbol{\beta}}_M^{\mathrm{IMP}})}{\widehat{K}(T_i)} - \frac{1}{n^2}\sum_{i=1}^n \frac{\Delta_i^{\mathrm{F}} D_i(\hat{\boldsymbol{\beta}}_{Q^{\mathrm{F}}}^{\mathrm{IMP}})}{\widehat{K}^{\mathrm{F}}(T_i^{\mathrm{F}})} \otimes \sum_{i=1}^n \frac{\Delta_i D_i(\hat{\boldsymbol{\beta}}_M^{\mathrm{IMP}})}{\widehat{K}(T_i)} \\
&\quad + \widehat{J}^{\mathrm{F}}\{D(\hat{\boldsymbol{\beta}}_{Q^{\mathrm{F}}}^{\mathrm{IMP}}) \otimes D(\hat{\boldsymbol{\beta}}_M^{\mathrm{IMP}})\} - \widehat{J}^{\mathrm{F}}\{D(\hat{\boldsymbol{\beta}}_{Q^{\mathrm{F}}}^{\mathrm{IMP}}) \otimes D(\hat{\boldsymbol{\beta}}_M^{\mathrm{IMP}}, u)\} \\
&\quad - \widehat{J}^{\mathrm{F}}\{D(\hat{\boldsymbol{\beta}}_{Q^{\mathrm{F}}}^{\mathrm{IMP}}, u) \otimes D(\hat{\boldsymbol{\beta}}_M^{\mathrm{IMP}})\} + \widehat{J}^{\mathrm{F}}\{D(\hat{\boldsymbol{\beta}}_{Q^{\mathrm{F}}}^{\mathrm{IMP}}, u) \otimes D(\hat{\boldsymbol{\beta}}_M^{\mathrm{IMP}}, u)\}. \qquad (2)
\end{aligned}
$$

The SW estimator for $I_1^{MQ^{\mathrm{F}}}$ is just the first three terms in Equation (2), with $\hat{\boldsymbol{\beta}}_{Q^{\mathrm{F}}}^{\mathrm{IMP}}$ replaced by $\hat{\boldsymbol{\beta}}_{Q^{\mathrm{F}}}^{\mathrm{SW}}$ and $\hat{\boldsymbol{\beta}}_{M}^{\mathrm{IMP}}$ replaced by $\hat{\boldsymbol{\beta}}_{M}^{\mathrm{SW}}$. When using event-free YOL as the effectiveness measure, the SW estimator for $I_1^{MX^{\mathrm{F}}}$ is the same as the SW estimator for $I_1^{MQ^{\mathrm{F}}}$ by replacing $\hat{\boldsymbol{\beta}}_{Q^{\mathrm{F}}}^{\mathrm{SW}}$ with $\hat{\boldsymbol{\beta}}_{X^{\mathrm{F}}}^{\mathrm{SW}}$, while the IMP estimator for $I_1^{MX^{\mathrm{F}}}$ consists of the first four terms in Equation (2) with $\hat{\boldsymbol{\beta}}_{Q^{\mathrm{F}}}^{\mathrm{IMP}}$ replaced by $\hat{\boldsymbol{\beta}}_{X^{\mathrm{F}}}^{\mathrm{SW}}$.

Note: Although the second term in Equation (2) can be omitted under large sample size conditions, our finite-sample simulation studies demonstrate that retaining this term leads to smaller differences between ECV and SCV while there is no notable impact on coverage probability (CP). Therefore, we recommend retaining the second term in Equation (2).

A.2.1: Variance estimator for data with different censoring times

The explicit formula for the IMP estimator of the regression coefficients for the early censored cost (M_0) from Section 2.4.2 of the main text is $\hat{\boldsymbol{\beta}}_{M_0}^{\mathrm{IMP}} = (\mathbf{B}_1^{M_0})^{-1}\mathbf{B}_2^{M_0}$, where

$$\mathbf{B}_1^{M_0} = \sum_{i=1}^{n}\left\{\frac{\Delta_{0i}}{\widehat{K}_0(T_i)} + \int_0^L \frac{\mathrm{d}N_i^{C_0}(u)}{\widehat{K}_0(u)}\right\}\boldsymbol{Z}_i^{M_0\otimes 2} - \sum_{i=1}^{n}\int_0^L \frac{\sum_{j=1}^{n} Y_{0j}(u)\boldsymbol{Z}_j^{M_0\otimes 2}}{\widehat{K}_0(u)Y_0(u)}\mathrm{d}N_i^{C_0}(u),$$
$$\mathbf{B}_2^{M_0} = \sum_{i=1}^{n}\left\{\frac{\Delta_{0i}M_{0i}}{\widehat{K}_0(T_i)} + \int_0^L \frac{M_{0i}(u)}{\widehat{K}_0(u)}\mathrm{d}N_i^{C_0}(u)\right\}\boldsymbol{Z}_i^{M_0} - \sum_{i=1}^{n}\int_0^L \frac{\sum_{j=1}^{n} M_{0j}(u)Y_{0j}(u)\boldsymbol{Z}_j^{M_0}}{\widehat{K}_0(u)Y_0(u)}\mathrm{d}N_i^{C_0}(u),$$

and the at-risk process for early censored cost is $Y_0(u) = \sum_{i=1}^n Y_{0i}(u) = \sum_{i=1}^n I(X_{0i} \geq u)$. The asymptotic variance-covariance matrix of early censored cost $\hat{\boldsymbol{\beta}}_{M_0}^{\mathrm{SW}}$ can be estimated by

$$\widehat{\mathrm{Var}}(\hat{\boldsymbol{\beta}}_{M_0}^{\mathrm{SW}}) = \frac{1}{n}(\hat{I}_0^{M_0})^{-1}\hat{I}_1^{M_0}(\hat{I}_0^{M_0})^{-1}, \tag{3}$$

where $\hat{I}_0^{M_0} = \frac{1}{n}\sum_{i=1}^n \boldsymbol{Z}_i^{M_0\otimes 2}$,

$$\hat{I}_1^{M_0} = \frac{1}{n}\sum_{i=1}^{n}\frac{\Delta_{0i}}{\widehat{K}_0(T_i)}\{D_i(\hat{\boldsymbol{\beta}}_{M_0}^{\mathrm{SW}})\}^{\otimes 2} + \widehat{J}_0\{D(\hat{\boldsymbol{\beta}}_{M_0}^{\mathrm{SW}})\otimes D(\hat{\boldsymbol{\beta}}_{M_0}^{\mathrm{SW}})\},$$
$$\widehat{J}_0(X\otimes Y) = \frac{1}{n}\int_0^L\left\{\widehat{G}_0(X\otimes Y, u) - \widehat{G}_0(X,u)\otimes\widehat{G}_0(Y,u)\right\}\frac{\mathrm{d}N^{C_0}(u)}{\widehat{K}_0(u)^2},$$

$\widehat{G}_0(W,u) = \frac{1}{n\widehat{S}_0(u)}\sum_{i=1}^n \frac{\Delta_{0i}}{\widehat{K}_0(T_i)} W_i I(T_i \geq u)$, $N^{C_0}(u) = \sum_{i=1}^n N_i^{C_0}(u) = \sum_{i=1}^n I(X_{0i} \leq u, \Delta_{0i} = 0)$, and $\widehat{S}_0(u)$ is the KM estimator for survival function of T_i at time u, $S_0(u) = \Pr(T_i > u)$, using data $(X_{0i}, \Delta_{0i}, i = 1, \ldots, n)$. The asymptotic variance-covariance estimator for $\hat{\boldsymbol{\beta}}_{M_0}^{\text{IMP}}$ takes the same formula (3) by replacing $\hat{\boldsymbol{\beta}}_{M_0}^{\text{SW}}$ with $\hat{\boldsymbol{\beta}}_{M_0}^{\text{IMP}}$ and set

$$\begin{aligned}
\hat{I}_1^{M_0} &= \frac{1}{n}\sum_{i=1}^n \frac{\Delta_{0i}}{\widehat{K}_0(T_i)}\{D_i(\hat{\boldsymbol{\beta}}_{M_0}^{\text{IMP}})\}^{\otimes 2} \\
&+\widehat{J}_0\{D(\hat{\boldsymbol{\beta}}_{M_0}^{\text{IMP}}) \otimes D(\hat{\boldsymbol{\beta}}_{M_0}^{\text{IMP}})\} + \widehat{J}_0\{D(\hat{\boldsymbol{\beta}}_{M_0}^{\text{IMP}}, u) \otimes D(\hat{\boldsymbol{\beta}}_{M_0}^{\text{IMP}}, u)\} \\
&-\widehat{J}_0\{D(\hat{\boldsymbol{\beta}}_{M_0}^{\text{IMP}}) \otimes D(\hat{\boldsymbol{\beta}}_{M_0}^{\text{IMP}}, u)\} - \widehat{J}_0\{D(\hat{\boldsymbol{\beta}}_{M_0}^{\text{IMP}}, u) \otimes D(\hat{\boldsymbol{\beta}}_{M_0}^{\text{IMP}})\}.
\end{aligned}$$

Similar to $\widehat{J}$, if $\widehat{J}_0$ involves $D(\hat{\boldsymbol{\beta}}_{M_0}^{\text{IMP}}, u)$, $\widehat{G}_0$ is replaced by $\widehat{G}_0^\star(W,u) = \sum_{i=1}^n W_i Y_{0i}(u)/Y_0(u)$.

A.2.2: Covariance estimator for data with different censoring times

Consider the martingale process for the censoring variable of cost C_{0i}: $\mathcal{M}_i^{C_0}(u) = N_i^{C_0}(u) - \int_0^u \lambda^{C_0}(t) Y_{0i}(t)\mathrm{d}t$, where $\lambda^{C_0}(t)$ is the hazard function for C_0, $\lambda^{C_0}(t) = \lim_{h\to 0}\frac{1}{h}\Pr(t < C_{0i} < t + h | C_{0i} \geq t, T \geq t)$. We define $\lambda^{CC_0}(u) = \lim_{h\to 0}\frac{1}{h}\Pr(t < C_0 = C < t + h | C_0 = C \geq t, T \geq t)$. From Wang and Zhao (2006), $\text{Cov}\{d\mathcal{M}_i^C(u), d\mathcal{M}_i^{C_0}(u)\} = Y_{0i}(u)\lambda^{CC_0}(u)du$. To derive Equation (9) in the main text, we have

$$\begin{aligned}
I_1^{M_0 Q} &= \text{E}\{D_i(\boldsymbol{\beta}_Q) \otimes D_i(\boldsymbol{\beta}_{M_0})\} + \text{E}\{W_1(\boldsymbol{\beta}_Q) \otimes W_1^{C_0}(\boldsymbol{\beta}_{M_0})\} - \text{E}\{W_1(\boldsymbol{\beta}_Q) \otimes W_2^{C_0}(\boldsymbol{\beta}_{M_0})\} \\
&-\text{E}\{W_2(\boldsymbol{\beta}_Q) \otimes W_1^{C_0}(\boldsymbol{\beta}_{M_0})\} + \text{E}\{W_2(\boldsymbol{\beta}_Q) \otimes W_2^{C_0}(\boldsymbol{\beta}_{M_0})\}, \qquad (4)
\end{aligned}$$

where

$$\begin{aligned}
W_1(\boldsymbol{\beta}_Q) &= \int_0^L [D_i(\boldsymbol{\beta}_Q) - G\{D(\boldsymbol{\beta}_Q), u\}]\frac{\mathrm{d}\mathcal{M}_i^C(u)}{K(u)}, \\
W_2(\boldsymbol{\beta}_Q) &= \int_0^L [D_i(\boldsymbol{\beta}_Q, u) - G\{D(\boldsymbol{\beta}_Q, u), u\}]\frac{\mathrm{d}\mathcal{M}_i^C(u)}{K(u)}, \\
W_1^{C_0}(\boldsymbol{\beta}_{M_0}) &= \int_0^L [D_i(\boldsymbol{\beta}_{M_0}) - G_0\{D(\boldsymbol{\beta}_{M_0}), u\}]\frac{\mathrm{d}\mathcal{M}_i^{C_0}(u)}{K_0(u)}, \\
W_2^{C_0}(\boldsymbol{\beta}_{M_0}) &= \int_0^L [D_i(\boldsymbol{\beta}_{M_0}, u) - G_0\{D(\boldsymbol{\beta}_{M_0}, u), u\}]\frac{\mathrm{d}\mathcal{M}_i^{C_0}(u)}{K_0(u)}.
\end{aligned}$$

Next,

$$\begin{aligned}
&\mathrm{E}\{W_1(\boldsymbol{\beta}_Q) \otimes W_1^{C_0}(\boldsymbol{\beta}_{M_0})\} \\
&= \mathrm{E}\int_0^L [D_i(\boldsymbol{\beta}_Q) - G\{D(\boldsymbol{\beta}_Q), u\}][D_i(\boldsymbol{\beta}_{M_0}) - G_0\{D(\boldsymbol{\beta}_{M_0}), u\}]\frac{Y_{0i}(u)\lambda^{CC_0}(u)}{K_0(u)K(u)}du \\
&= \mathrm{E}\int_0^L [D_i(\boldsymbol{\beta}_Q)D_i(\boldsymbol{\beta}_{M_0}) - G\{D(\boldsymbol{\beta}_Q), u\}D_i(\boldsymbol{\beta}_{M_0}) - D_i(\boldsymbol{\beta}_Q)G_0\{D(\boldsymbol{\beta}_{M_0}), u\} \\
&\qquad + G\{D(\boldsymbol{\beta}_Q), u\} \otimes G_0\{D(\boldsymbol{\beta}_{M_0}), u\}]I(T_i \geq u)\frac{\lambda^{CC_0}(u)}{K(u)}du \\
&= \int_0^L [G_0\{D(\boldsymbol{\beta}_Q) \otimes D(\boldsymbol{\beta}_{M_0}), u\} - G_0\{D(\boldsymbol{\beta}_Q), u\} \otimes G_0\{D(\boldsymbol{\beta}_{M_0}), u\}]S(u)\frac{\lambda^{CC_0}(u)}{K(u)}du,
\end{aligned}$$

which can be estimated by $\widehat{J}^{C_0}\{D(\boldsymbol{\beta}_Q) \otimes D(\boldsymbol{\beta}_{M_0})\}$. The last three terms in Equation (4) can be derived similarly. Hence, the IMP estimator for $I_1^{M_0Q}$ is

$$\begin{aligned}
\hat{I}_1^{M_0Q} \;=\; & \frac{1}{n}\sum_{i=1}^n \frac{\Delta_{0i}D_i(\hat{\boldsymbol{\beta}}_Q^{\text{IMP}}) \otimes D_i(\hat{\boldsymbol{\beta}}_{M_0}^{\text{IMP}})}{\widehat{K}_0(T_i)} - \frac{1}{n^2}\sum_{i=1}^n \frac{\Delta_i D_i(\hat{\boldsymbol{\beta}}_Q^{\text{IMP}})}{\widehat{K}(T_i)} \otimes \sum_{i=1}^n \frac{\Delta_{0i}D_i(\hat{\boldsymbol{\beta}}_{M_0}^{\text{IMP}})}{\widehat{K}_0(T_i)} \\
& + \widehat{J}^{C_0}\{D(\hat{\boldsymbol{\beta}}_Q^{\text{IMP}}) \otimes D(\hat{\boldsymbol{\beta}}_{M_0}^{\text{IMP}})\} - \widehat{J}^{C_0}\{D(\hat{\boldsymbol{\beta}}_Q^{\text{IMP}}) \otimes D(\hat{\boldsymbol{\beta}}_{M_0}^{\text{IMP}}, u)\} \\
& - \widehat{J}^{C_0}\{D(\hat{\boldsymbol{\beta}}_Q^{\text{IMP}}, u) \otimes D(\hat{\boldsymbol{\beta}}_{M_0}^{\text{IMP}})\} + \widehat{J}^{C_0}\{D(\hat{\boldsymbol{\beta}}_Q^{\text{IMP}}, u) \otimes D(\hat{\boldsymbol{\beta}}_{M_0}^{\text{IMP}}, u)\}. \qquad (5)
\end{aligned}$$

Similarly, the SW estimator for $I_1^{M_0Q}$ is simply the first three terms in Equation (5), with $\hat{\boldsymbol{\beta}}_Q^{\text{IMP}}$ replaced by $\hat{\boldsymbol{\beta}}_Q^{\text{SW}}$ and $\hat{\boldsymbol{\beta}}_{M_0}^{\text{IMP}}$ replaced by $\hat{\boldsymbol{\beta}}_{M_0}^{\text{SW}}$. When using YOL as the effectiveness measure, the SW estimator for $I_1^{M_0X}$ is the same as the SW estimator for $I_1^{M_0Q}$ by replacing $\hat{\boldsymbol{\beta}}_Q^{\text{SW}}$ with $\hat{\boldsymbol{\beta}}_X^{\text{SW}}$, while the IMP estimator for $I_1^{M_0X}$ consists of the first four terms in Equation (5), with $\hat{\boldsymbol{\beta}}_Q^{\text{IMP}}$ replaced by $\hat{\boldsymbol{\beta}}_X^{\text{SW}}$. The second term in Equation (5) remains for the same reason as in the scenario with different terminating events.

Web Appendix B: Additional numerical results for ICER

Web Table 1 presents the true mean values of effectiveness (years) and cost (\$1000) between different treatments and subgroups for the simulated data discussed in Section 3 of the main text. The mathematical derivation is provided in Web Appendix E. Utilizing these values,

we can derive the true ICER for each subgroup by computing the ratio of the mean difference in costs to the mean difference in effectiveness. The true INB can be obtained by plugging different willingness-to-pay (WTP) values into the INB formula $\text{INB}(\lambda) = \lambda \cdot \Delta\text{Effect} - \Delta\text{Cost}$.

Web Table 1: True values of mean effectiveness and costs stratified by treatments and subgroups in simulated data.

Measurement	Subgroup U	Treatment $A = 0$	Treatment $A = 1$	Mean difference between treatment 1 and 0 (Δ)
HF-free survival time	0	3.33	1.74	-1.59
	1	2.25	6.55	4.30
HF-free QAL	0	2.16	1.65	-0.51
	1	1.46	6.22	4.76
Overall survival time	0	6.04	5.19	-0.86
	1	4.59	7.06	2.46
Overall QAL	0	3.93	4.93	1.00
	1	2.99	6.70	3.72
Cost (in $1000)	0	8.72	13.54	4.83
	1	6.45	9.10	2.64

B.1: Additional results for ICER using data with different terminating events

We evaluate the performance of bootstrap CIs and compare it with our methods. In our simulation, the bootstrap samples are distributed across the northeast and northwest regions of the cost-effectiveness (CE) plane for subgroup $U = 0$, while the majority of them from subgroup $U = 1$ lie in the northeast region of the CE plane. Therefore, we consider two methods for constructing the Bootstrap CIs. The first is the bootstrap percentile method, which sorts the bootstrap ICERs from the smallest to the largest and obtains the $100(1 - 2\alpha)\%$ CI using the upper and lower $100\alpha\%$ cut-off points. The other one is the reordered bootstrap method proposed by Wang and Zhao (2008), which rearranges the ICER bootstrap samples according to their natural order on the CE plane before selecting the endpoints of the CIs. Web Table 2 shows the comparison of the three methods with HF-free QAL as the effectiveness measure. The bootstrap percentile method has poor performance in the

subgroup $U = 0$, with much higher CPs than the nominal levels. This issue arises in ICER because the percentile method always yields finite intervals, which can be misleading when the difference in effectiveness between two groups is not significant. In our simulation, the effectiveness difference for subgroup $U = 0$ is close to zero, inducing extreme ICER instability and failure of the bootstrap percentile method, as the bootstrapped ICER replicates span multiple quadrants of the CE plane. In all simulation replications under this setting, the discriminant of Equation (7) in the main text for constructing Fieller's CIs is positive. When the denominator of Equation (7) in the main text is positive, the Fieller-based CI is a finite interval between the two roots. When the denominator is negative, the method yields a disjoint confidence set, defined as the complement of the finite interval between the roots. This construction is consistent with the theoretical properties of Fieller's method discussed in Section 2.3.3 of the main text. For subgroup $U = 1$, the CPs of the bootstrap percentile method closely align with those of the reordered bootstrap method, as the effectiveness is significantly larger than zero. Overall, the performance of the reordered bootstrap is comparable with our methods, with both achieving CPs close to the nominal levels. However, our method shows superior computational efficiency compared to two bootstrap methods.

Web Table 2: Summary of empirical coverage probabilities of CIs for ICERs using HF-free QAL as the effectiveness measure, based on data with different terminating events from 2000 simulations with a sample size of 400.

			Light censoring			Heavy censoring		
Nominal level	Subgroup U	Method	New	Percentile	Reordered	New	Percentile	Reordered
95%	0	SW	0.942	0.979	0.951	0.926	0.971	0.938
		IMP	0.947	0.977	0.952	0.942	0.975	0.950
	1	SW	0.939	0.943	0.943	0.938	0.930	0.932
		IMP	0.946	0.942	0.942	0.941	0.941	0.947
90%	0	SW	0.896	0.955	0.906	0.887	0.945	0.886
		IMP	0.891	0.948	0.893	0.889	0.947	0.896
	1	SW	0.885	0.890	0.890	0.888	0.881	0.881
		IMP	0.896	0.899	0.899	0.893	0.886	0.890
80%	0	SW	0.803	0.896	0.808	0.783	0.890	0.789
		IMP	0.785	0.892	0.808	0.783	0.891	0.797
	1	SW	0.786	0.791	0.791	0.794	0.766	0.766
		IMP	0.785	0.783	0.783	0.790	0.794	0.796

Notes: New is our proposed method; percentile is the naive bootstrap percentile method; reordered is the reordered bootstrap percentile method; SW denotes the simple weighted estimator for both costs and HF-free QAL; IMP refers to the improved estimator for both costs and HF-free QAL; the number of bootstrap replications is 1000.

The results using HF-free YOL as the effectiveness measure under the scenario of different terminating events (main text Section 3.1) are presented in Web Table 3 and Web Table 4.

Web Table 3: Summary of simulation results for ICER estimation, including median bias, covariance between costs and HF-free YOL, empirical coverage probabilities of 95% CIs, and median CI angle under different terminating events from 2000 simulations.

			Light censoring					Heavy censoring				
n	U	Method	Bias	SCV	ECV	CP	Angle	Bias	SCV	ECV	CP	Angle
400	0	Unadjusted	5.821	13	14	0.000	0.368	5.766	36	30	0.009	0.503
		Subgroup	-0.061	85	92	0.946	0.376	0.036	143	127	0.929	0.477
		SW	-0.046	91	96	0.943	0.389	-0.048	149	151	0.931	0.517
		IMP	-0.029	110	110	0.943	0.406	-0.023	172	171	0.935	0.533
	1	Unadjusted	2.165	13	14	0.000	0.368	2.110	36	30	0.008	0.503
		Subgroup	-0.002	65	72	0.943	0.227	0.004	109	105	0.939	0.305
		SW	-0.000	74	72	0.943	0.227	0.002	109	108	0.935	0.309
		IMP	-0.001	85	84	0.945	0.249	0.006	133	132	0.941	0.322
800	0	Unadjusted	5.760	7	7	0.000	0.262	5.765	16	16	0.000	0.355
		Subgroup	-0.001	48	46	0.949	0.265	0.006	71	68	0.942	0.333
		SW	0.012	52	48	0.946	0.273	0.014	88	79	0.935	0.364
		IMP	0.015	58	55	0.948	0.284	0.016	96	88	0.943	0.368
	1	Unadjusted	2.103	7	7	0.000	0.262	2.109	16	16	0.000	0.355
		Subgroup	0.000	35	36	0.949	0.159	0.002	50	52	0.943	0.216
		SW	0.001	35	36	0.944	0.160	0.003	51	54	0.936	0.219
		IMP	0.001	42	42	0.958	0.176	0.002	67	66	0.957	0.227
1200	0	Unadjusted	5.786	3	5	0.000	0.212	5.773	9	11	0.000	0.286
		Subgroup	-0.030	30	31	0.950	0.216	0.001	45	45	0.944	0.270
		SW	-0.023	28	32	0.949	0.221	-0.021	56	52	0.942	0.292
		IMP	-0.032	35	37	0.949	0.229	-0.015	64	58	0.941	0.297
	1	Unadjusted	2.130	3	5	0.000	0.212	2.117	9	11	0.000	0.286
		Subgroup	0.001	25	24	0.951	0.130	0.002	35	35	0.946	0.176
		SW	0.000	25	24	0.951	0.130	0.001	38	36	0.953	0.178
		IMP	0.002	28	28	0.958	0.143	0.002	45	44	0.950	0.184

Notes: U denotes the subgroup; Bias is the difference between the true ICER (-3.04 for $U = 0$ and 0.61 for $U = 1$, in \$1000/year) and the median of estimates from 2000 simulations; SCV is the sample covariance between the mean cost estimators and the mean HF-free YOL estimators from 2000 simulations; ECV is the average of the estimated covariances obtained by our method; CP is the proportion containing the true ICER within the 95% CI; Angle is the median wedge angle of the CIs; SW denotes the simple weighted estimator for both costs and HF-free YOL; IMP refers to the improved estimator for costs only; Subgroup represents the separate simple weighted analysis within $U = 0$ and $U = 1$; Unadjusted represents simple weighted analysis without adjustment for covariate U.

Web Table 4: Summary of empirical coverage probabilities of CIs for ICERs using HF-free YOL as the effectiveness measure, based on data with different terminating events from 2000 simulations with a sample size of 400.

			Light censoring			Heavy censoring		
Nominal level	Subgroup U	Method	New	Percentile	Reordered	New	Percentile	Reordered
95%	0	SW	0.943	0.963	0.948	0.931	0.968	0.939
		IMP	0.943	0.962	0.947	0.935	0.973	0.941
	1	SW	0.943	0.942	0.942	0.935	0.935	0.934
		IMP	0.945	0.938	0.939	0.941	0.945	0.950
90%	0	SW	0.901	0.918	0.905	0.884	0.927	0.889
		IMP	0.897	0.920	0.906	0.881	0.929	0.883
	1	SW	0.891	0.891	0.891	0.886	0.889	0.890
		IMP	0.891	0.889	0.889	0.893	0.893	0.897
80%	0	SW	0.797	0.809	0.802	0.780	0.839	0.797
		IMP	0.791	0.811	0.805	0.777	0.839	0.794
	1	SW	0.785	0.788	0.788	0.790	0.778	0.777
		IMP	0.790	0.797	0.797	0.791	0.794	0.795

Notes: New is our proposed method; percentile is the naive bootstrap percentile method; reordered is the reordered bootstrap percentile method; SW denotes the simple weighted estimator for both costs and HF-free YOL; IMP refers to the improved estimator for costs only; the number of bootstrap replications is 1000.

We further investigate a simulation scenario involving multiple covariates and confounding, with $n = 1200$ under heavy censoring. Here, two covariates are generated as $U_1 \sim$ Bernoulli(0.5) and $U_2 \sim$ Bernoulli(0.35), with treatment assignment A following the logistic model $\text{logit}\{P(A = 1|U_2)\} = -1.5 + 1.5U_2$. Survival times to death and HF event are generated from exponential distributions with rate parameters $1/\exp(2.2 - 0.3A - 0.5U_1 + 1.2A \times U_1 + U_2 + 1.5A \times U_2)$ and $1/\exp\{0.8 \times (2.2 - 0.5U) + 3 \times (-0.3A + 1.2A \times U_1) + U_2 + 1.5A \times U_2\}$, respectively. Web Table 5 compares the results of our proposed method with subgroup analysis for estimating ICERs in subgroup U_1. Our model incorporates the treatment indicator

A, covariates U_1 and U_2, and all possible interaction terms. The subgroup approach performs analyses within $U_1 = 0$ and $U_1 = 1$ separately, failing to adjust for the additional confounder U_2. The proposed method demonstrates significantly lower bias and higher CPs than the subgroup approach. Furthermore, the IMP method outperforms the SW method across all metrics, including bias, CP, and CI wedge angle.

Web Table 5: Summary of simulation results for ICER estimation using HF-free QAL with an additional confounder, under different terminating events from 2000 simulations with a sample size of 1200 and heavy censoring.

U_1	Method	Bias	SCV	ECV	CP	Angle
0	Subgroup	-3.736	162	159	0.105	0.201
	SW	-0.030	60	55	0.946	0.174
	IMP	0.008	126	119	0.957	0.170
1	Subgroup	-0.019	33	32	0.918	0.140
	SW	0.006	63	55	0.926	0.174
	IMP	0.001	128	120	0.950	0.167

Notes: U_1 denotes the subgroup; Bias is the difference between the true ICER (6.63 for $U_1 = 0$ and 0.45 for $U_1 = 1$, in \$1000/year) and the median of estimates from 2000 simulations; SCV is the sample covariance between the mean cost estimators and the mean HF-free QAL estimators from 2000 simulations; ECV is the average of the estimated covariances obtained by our method; CP is the proportion containing the true ICER within the 95% CI; Angle is the median wedge angle of the CIs; our model incorporates the treatment indicator A, covariates U_1 and U_2, and all possible interaction terms; SW denotes the simple weighted estimator for both costs and HF-free QAL; IMP refers to the improved estimator for both costs and HF-free QAL; Subgroup represents the separate simple weighted analysis within $U_1 = 0$ and $U_1 = 1$ without adjustment for covariate U_2.

The results for the MADIT-CRT data using unrestricted QAL, HF-free YOL, and unrestricted YOL as effectiveness measures (main text Section 4.1) are summarized in Web Table 6 and Web Table 7. Web Figure 1 presents the bootstrap samples from the IMP method, along with ICER estimates and 95% CIs from both the IMP and Bootstrap methods using HF-free YOL as the effectiveness measure.

Web Table 6: Estimated mean total costs ($1000), unrestricted QAL (year), ICERs ($1000/year), and CIs, limited to a 4-year time horizon, for LBBB and non-LBBB groups in the MADIT-CRT data with different terminating events.

	CRT–ICD Estimate (SE)		ICD Estimate (SE)		Difference Estimate (SE)	
	SW	IMP	SW	IMP	SW	IMP
LBBB group						
Cost	55.5 (2.63)	62.3 (1.78)	56.5 (4.32)	59.6 (1.89)	-1.0 (5.06)	2.7 (2.67)
Unrestricted QAL	3.2 (0.02)	3.3 (0.03)	3.0 (0.04)	3.2 (0.04)	0.2 (0.12)	0.1 (0.16)
Non-LBBB group						
Cost	64.1 (3.07)	67.2 (2.79)	62.6 (8.48)	57.4 (3.45)	1.4 (9.02)	9.8 (4.50)
Unrestricted QAL	2.8 (0.03)	2.8 (0.04)	3.1 (0.05)	2.9 (0.06)	-0.2 (0.16)	-0.2 (0.21)

	LBBB group		Non-LBBB group	
Method	ECV	ICER (95% CI)	ECV	ICER (95% CI)
Subgroup	-0.122	-4.3 (-62.9, 92.9)	-0.827	-4.4 $(-\infty, +\infty)$
SW	-0.143	-4.2 $(-105.3, 327.8)$	-0.529	-6.3 $(-\infty, +\infty)$
IMP	0.109	22.6 $(-\infty, +\infty)$	0.481	59.9 $(-\infty, -2.4) \cup (48.4 + \infty)$

Notes: SE is estimated standard error; ECV is the estimated covariance between cost and effectiveness; SW denotes the simple weighted estimator; IMP refers to the improved estimator; Our methods include treatment, LBBB status, and their interaction in the model; Subgroup refers to subgroup simple weighted analysis within LBBB and non-LBBB groups.

Web Table 7: Estimated mean total costs ($1000), HF-free YOL (Year), Unrestricted YOL (Year), ICERs ($1000/year), and CIs, limited to a 4-year time horizon, for LBBB and non-LBBB groups in the MADIT-CRT data with different terminating events.

	CRT–ICD Estimate (SE)		ICD Estimate (SE)		Difference Estimate (SE)	
	SW	IMP	SW	IMP	SW	IMP
LBBB group						
Cost	55.5 (2.63)	62.3 (1.78)	56.5 (4.32)	59.6 (1.89)	-1.0 (5.06)	2.7 (2.67)
HF-free YOL	3.4 (0.02)	–	3.0 (0.04)	–	0.4 (0.14)	–
Unrestricted YOL	3.7 (0.01)	–	3.5 (0.02)	–	0.1 (0.06)	
Non-LBBB group						
Cost	64.1 (3.07)	67.2 (2.79)	62.6 (8.48)	57.4 (3.45)	1.4 (9.02)	9.8 (4.50)
HF-free YOL	2.9 (0.05)	–	3.3 (0.05)	–	-0.4 (0.21)	–
Unrestricted YOL	3.4 (0.03)	–	3.6 (0.02)	–	-0.3 (0.09)	

Effectiveness measure: HF-free YOL

	LBBB group		Non-LBBB group	
Method	ECV	ICER (95% CI)	ECV	ICER (95% CI)
Subgroup	-0.247	2.4 (-22.1, 30.0)	-0.598	-2.3 $(-\infty, +\infty)$
SW	-0.276	-2.3 $(-24.5, 36.0)$	-0.448	-3.6 $(-336.1, -\infty) \cup (-227.3, -\infty)$
IMP	-0.005	6.3 $(-6.6, 27.6)$	0.037	-24.5 $(-\infty, -2.2) \cup (1995.0, +\infty)$
Bootstrap-SW	–	$(-26.4, 35.2)$	–	$(-85.3, -\infty) \cup (-133.8, -\infty)$
Bootstrap-IMP	–	$(-7.8, 32.1)$	–	$(-\infty, -0.3) \cup (88.0, +\infty)$

Effectiveness measure: Unrestricted YOL

	LBBB group		Non-LBBB group	
Method	ECV	ICER (95% CI)	ECV	ICER (95% CI)
Subgroup	-0.039	-7.7 (-130.6, 132.3)	-0.104	-4.0 (-188.5, 221.2)
SW	-0.043	-7.7 $(-217.9, 264.9)$	-0.074	-5.7 $(-103.8, 98.2)$
IMP	0.018	20.8 $(-33.4, 357.6)$	0.056	-38.4 $(-174.6, -3.3)$

Notes: SE is estimated standard error; ECV is the estimated covariance between cost and effectiveness; SW denotes the simple weighted estimator; IMP refers to the improved estimator; Our methods include treatment, LBBB status, and their interaction in the model; Subgroup refers to subgroup simple weighted analysis within LBBB and non-LBBB groups; Bootstrap denotes the angle-based bootstrap method with 1000 bootstrap replications.

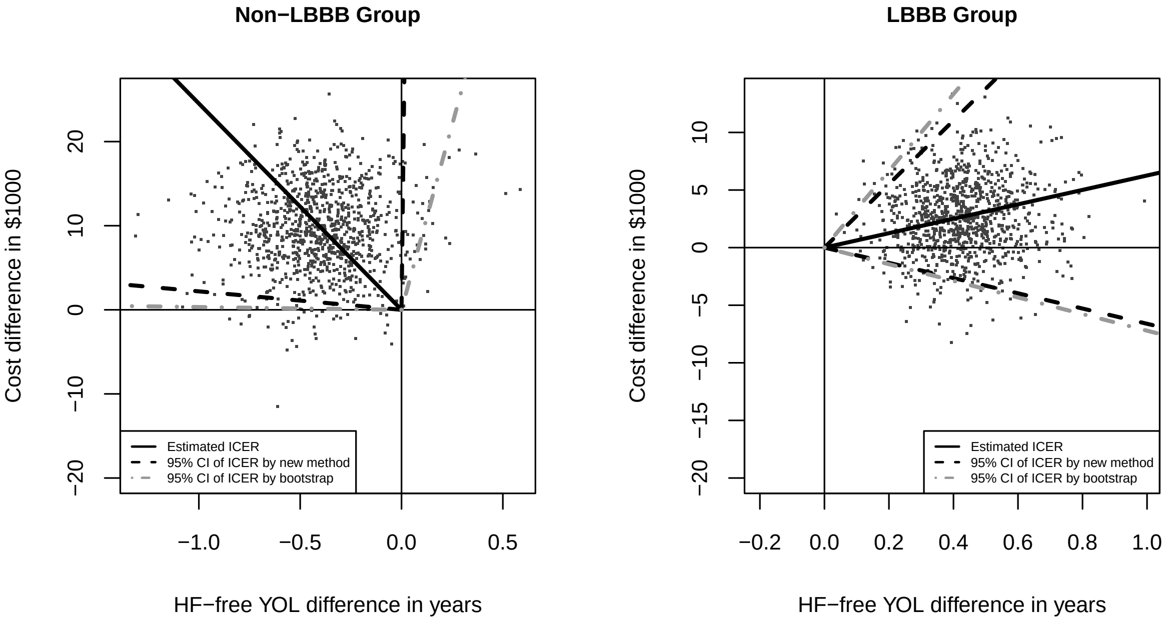

Web Figure 1: Estimated ICERs and 95% CIs using HF-free YOL as the effectiveness measure for LBBB and non-LBBB groups in the MADIT-CRT study, limited to a 4-year time horizon. The dots represent 1000 bootstrap samples. The solid lines denote the estimated ICERs; the black dashed lines and the gray dot-dashed lines indicate CI limits obtained by IMP method and the bootstrap method, respectively.

B.2: Additional results for ICER using data with different censoring times

Additional simulation results for the scenario with different censoring times (main text Section 3.2) are provided in Web Table 8 and Web Table 9. These tables compare the proposed method with the naive method of censoring survival time early in terms of estimation efficiency and the bootstrap methods, respectively.

Web Table 8: Summary of sample standard errors of the estimators from the proposed methods and naive method, based on data with different censoring times from 2000 simulations.

	$n = 400$		$n = 800$		$n = 1200$	
SSE of Effectiveness	$U = 0$	$U = 1$	$U = 0$	$U = 1$	$U = 0$	$U = 1$
QAL-IMP						
New method	0.482	0.482	0.336	0.339	0.282	0.280
Naive method	0.662	0.684	0.473	0.486	0.387	0.401
YOL-SW						
New method	0.574	0.546	0.395	0.380	0.335	0.312
Naive method	0.879	0.810	0.590	0.559	0.491	0.454

Notes: SSE is the sample standard error; SW denotes the simple weighted estimator; IMP refers to the improved estimator; Naive method refers to censoring the survival time early; YOL is year of life estimated using the SW estimator; QAL is quality adjusted lifetime estimated using the IMP estimator to better incorporate the health history.

Web Table 9: Summary of empirical coverage probabilities of CIs for ICERs using QAL or YOL as the effectiveness measure, based on data with different censoring times from 2000 simulations with a sample size of 400.

Nominal level	Subgroup U	Method	QAL New	QAL Percentile	QAL Reordered	YOL New	YOL Percentile	YOL Reordered
95%	0	SW	0.944	0.971	0.947	0.943	0.975	0.954
		IMP	0.949	0.970	0.946	0.951	0.975	0.955
	1	SW	0.945	0.942	0.943	0.942	0.952	0.944
		IMP	0.954	0.947	0.958	0.947	0.950	0.950
90%	0	SW	0.894	0.947	0.905	0.899	0.945	0.897
		IMP	0.901	0.945	0.892	0.893	0.951	0.903
	1	SW	0.894	0.885	0.886	0.881	0.898	0.895
		IMP	0.900	0.890	0.898	0.893	0.895	0.897
80%	0	SW	0.799	0.887	0.804	0.798	0.896	0.796
		IMP	0.793	0.893	0.796	0.795	0.895	0.794
	1	SW	0.788	0.780	0.781	0.792	0.799	0.796
		IMP	0.793	0.787	0.790	0.795	0.789	0.789

Notes: New is our proposed method; percentile is the naive bootstrap percentile method; reordered is the reordered bootstrap percentile method; SW denotes the simple weighted estimator for early-censored costs, QAL, and YOL; IMP refers to the improved estimator for early-censored costs and QAL only; the number of bootstrap replications is 1000.

We evaluate a more complex scenario involving multiple covariates and confounding at $n = 1200$, using the same simulation setting and parameters detailed in Web Appendix B.1. Web Table 10 compares ICER estimates for subgroup U_1 using QAL as the effectiveness measure. Our model adjusts for treatment A, covariates U_1 and U_2, and all interactions, whereas the subgroup approach fails to account for U_2 and associated confounding. Consequently, our proposed method demonstrates significantly lower median bias and higher CPs.

Web Table 10: Summary of simulation results for ICER estimation using QAL with an additional confounder, under different censoring times from 2000 simulations with a sample size of 1200.

U_1	Method	Bias	SCV	ECV	CP	Angle
0	Subgroup	-0.720	107	110	0.188	0.118
	SW	-0.021	134	136	0.942	0.160
	IMP	-0.007	182	181	0.949	0.175
1	Subgroup	-0.038	30	28	0.902	0.163
	SW	0.003	45	39	0.947	0.206
	IMP	0.000	76	69	0.955	0.257

Notes: U_1 denotes the subgroup; Bias is the difference between the true ICER (3.15 for $U_1 = 0$ and 0.58 for $U_1 = 1$, in \$1000/year) and the median of estimates from 2000 simulations; SCV is the sample covariance between the mean cost estimators and the mean QAL estimators from 2000 simulations; ECV is the average of the estimated covariances obtained by our method; CP is the proportion containing the true ICER within the 95% CI; Angle is the median wedge angle of the CIs; our model incorporates the treatment indicator A, covariates U_1 and U_2, and all possible interaction terms; SW denotes the simple weighted estimator for both costs and QAL; IMP refers to the improved estimator for both costs and QAL; Subgroup represents the separate simple weighted analysis within $U_1 = 0$ and $U_1 = 1$ without adjustment for covariate U_2.

Web Appendix C: Additional numerical results for INB

C.1: Additional results for INB using data with different terminating events

This subsection presents the additional numerical results for the estimated INB using data with different terminating events, in both simulations (main text Section 3.1) and the MADIT-CRT example (main text Section 4.1). Web Table 11 and Web Table 12 summarize the simulation results of the estimated INBs based on 2000 simulation runs for different sample sizes, WTP, and levels of censoring with HF-free QAL and HF-free YOL as effectiveness measures. Both the SW method and the IMP method yield small biases and CPs close to

the nominal value. The estimated standard errors (ESE) of both estimators are close to the empirical standard errors (SEE), and they decrease with increasing sample size from 400 to 1200. Additionally, IMP estimators generally offer CPs closer to the nominal level 95%, particularly with smaller sample sizes. It is also observed that the IMP estimators produce smaller biases and standard errors compared to the SW estimators in heavy censoring cases. Therefore, selecting the IMP estimator is advantageous when cost history data is available, especially in scenarios with small sample sizes and heavy censoring.

Web Table 11: Summary of estimated INBs (in $1000) using HF-free QAL as the effectiveness measure with different WTP values (in $1000) and censoring rates, based on data with different terminating events from 2000 simulations.

				Light censoring		Heavy censoring	
n	WTP	Subgroup U	Method	Bias (SEE, ESE)	CP	Bias (SEE, ESE)	CP
400	1.5	0	SW	0.01 (0.70, 0.69)	0.941	0.02 (0.99, 0.96)	0.929
			IMP	0.01 (0.73, 0.72)	0.941	0.00 (0.90, 0.89)	0.939
		1	SW	0.00 (0.58, 0.56)	0.947	-0.02 (0.72, 0.71)	0.941
			IMP	0.00 (0.57, 0.57)	0.951	0.00 (0.67, 0.66)	0.941
	3	0	SW	0.01 (0.92, 0.90)	0.941	0.03 (1.27, 1.23)	0.931
			IMP	0.01 (0.90, 0.87)	0.936	0.00 (1.04, 1.01)	0.935
		1	SW	0.00 (1.15, 1.13)	0.941	-0.01 (1.38, 1.38)	0.947
			IMP	0.01 (1.18, 1.17)	0.947	0.00 (1.38, 1.36)	0.942
800	1.5	0	SW	0.01 (0.49, 0.49)	0.947	0.01 (0.68, 0.68)	0.944
			IMP	0.01 (0.50, 0.51)	0.953	0.01 (0.62, 0.63)	0.954
		1	SW	0.00 (0.40, 0.40)	0.943	-0.01 (0.51, 0.51)	0.945
			IMP	0.00 (0.40, 0.40)	0.950	0.00 (0.47, 0.47)	0.947
	3	0	SW	0.01 (0.64, 0.64)	0.947	0.01 (0.87, 0.87)	0.954
			IMP	0.01 (0.62, 0.62)	0.949	0.01 (0.71, 0.72)	0.950
		1	SW	0.01 (0.79, 0.80)	0.948	-0.01 (0.99, 0.98)	0.944
			IMP	0.01 (0.83, 0.83)	0.948	0.01 (0.98, 0.97)	0.950
1200	1.5	0	SW	-0.01 (0.41, 0.40)	0.948	0.01 (0.57, 0.55)	0.947
			IMP	-0.01 (0.42, 0.41)	0.947	-0.01 (0.53, 0.51)	0.938
		1.5	SW	0.00 (0.32, 0.33)	0.952	0.00 (0.41, 0.41)	0.952
			IMP	0.00 (0.32, 0.33)	0.955	0.00 (0.37, 0.38)	0.962
	3	0	SW	0.00 (0.53, 0.52)	0.942	0.02 (0.71, 0.71)	0.945
			IMP	-0.01 (0.51, 0.50)	0.939	0.00 (0.60, 0.59)	0.938
		1	SW	0.01 (0.65, 0.65)	0.953	0.00 (0.80, 0.80)	0.949
			IMP	0.01 (0.67, 0.68)	0.954	0.01 (0.77, 0.79)	0.960

Notes: WTP is decision maker's willingness-to-pay for an additional unit of effectiveness; Bias represents the absolute difference between the mean estimate and the true value; SEE indicates the empirical standard error of the estimates; ESE is the mean of the estimated standard errors; CP is the proportion containing the true INB within the 95% CI; SW denotes the simple weighted estimator for both costs and HF-free QAL; IMP refers to the improved estimator for both costs and HF-free QAL.

Web Table 12: Summary of estimated INBs (in \$1000) using HF-free YOL as the effectiveness measure with different WTP values (in \$1000) and censoring rates, based on data with different terminating events from 2000 simulations.

				Light censoring		Heavy censoring	
n	WTP	Subgroup U	Method	Bias (SEE, ESE)	CP	Bias (SEE, ESE)	CP
400	1.5	0	SW	0.01 (0.77, 0.75)	0.937	0.03 (1.07, 1.04)	0.929
			IMP	0.01 (0.80, 0.79)	0.936	0.03 (1.03, 1.02)	0.935
		1	SW	0.00 (0.64, 0.63)	0.944	0.00 (0.81, 0.80)	0.944
			IMP	0.00 (0.65, 0.63)	0.947	0.01 (0.79, 0.79)	0.945
	3	0	SW	0.02 (1.17, 1.13)	0.939	0.06 (1.59, 1.54)	0.924
			IMP	0.02 (1.17, 1.14)	0.936	0.06 (1.54, 1.51)	0.927
		1	SW	0.01 (1.32, 1.29)	0.942	0.01 (1.61, 1.60)	0.942
			IMP	0.01 (1.30, 1.28)	0.941	0.02 (1.58, 1.57)	0.938
800	1.5	0	SW	0.02 (0.53, 0.53)	0.945	0.01 (0.73, 0.74)	0.944
			IMP	0.02 (0.55, 0.56)	0.948	0.02 (0.71, 0.72)	0.952
		1	SW	0.00 (0.44, 0.45)	0.941	0.00 (0.58, 0.57)	0.943
			IMP	0.00 (0.44, 0.45)	0.950	0.00 (0.55, 0.56)	0.953
	3	0	SW	0.02 (0.81, 0.80)	0.946	0.02 (1.10, 1.09)	0.948
			IMP	0.03 (0.81, 0.81)	0.950	0.02 (1.07, 1.07)	0.947
		1	SW	0.01 (0.91, 0.91)	0.943	0.00 (1.15, 1.14)	0.938
			IMP	0.01 (0.90, 0.90)	0.946	0.00 (1.12, 1.12)	0.946
1200	1.5	0	SW	0.00 (0.44, 0.43)	0.945	0.01 (0.61, 0.60)	0.945
			IMP	0.00 (0.46, 0.46)	0.943	0.01 (0.59, 0.59)	0.944
		1	SW	0.00 (0.36, 0.36)	0.953	0.00 (0.47, 0.47)	0.950
			IMP	0.00 (0.36, 0.37)	0.956	0.00 (0.46, 0.46)	0.954
	3	0	SW	0.00 (0.67, 0.66)	0.943	0.03 (0.89, 0.89)	0.942
			IMP	0.00 (0.67, 0.66)	0.943	0.02 (0.86, 0.87)	0.945
		1	SW	0.01 (0.74, 0.75)	0.950	0.01 (0.93, 0.93)	0.952
			IMP	0.01 (0.73, 0.74)	0.949	0.00 (0.92, 0.91)	0.947

Notes: WTP is decision maker's willingness-to-pay for an additional unit of effectiveness; Bias represents the absolute difference between the mean estimate and the true value; SEE indicates the empirical standard error of the estimates; ESE is the mean of the estimated standard errors; CP is the proportion containing the true INB within the 95% CI; SW denotes the simple weighted estimator for both costs and HF-free YOL; IMP refers to the improved estimator for costs only.

The estimated INBs and the corresponding 95% CIs using the proposed IMP method for the MADIT-CRT data of both non-LBBB and LBBB groups are summarized in Web

Figure 2 and Web Figure 3, using HF-free QAL and HF-free YOL as effectiveness measures, respectively. The solid lines represent the INB estimates as a function of WTP, while the dashed lines indicate the corresponding 95% CIs. The horizontal intercepts represent the ICER estimates and the corresponding confidence limits, calculated using the Fieller method as described in the main text. For the non-LBBB group, the 95% lower limit of the INB never intersects the horizontal axis, implying that at a significance level of $\alpha = 0.05$, no positive WTP value would reject the null hypothesis $\text{INB}(\lambda) \leq 0$.

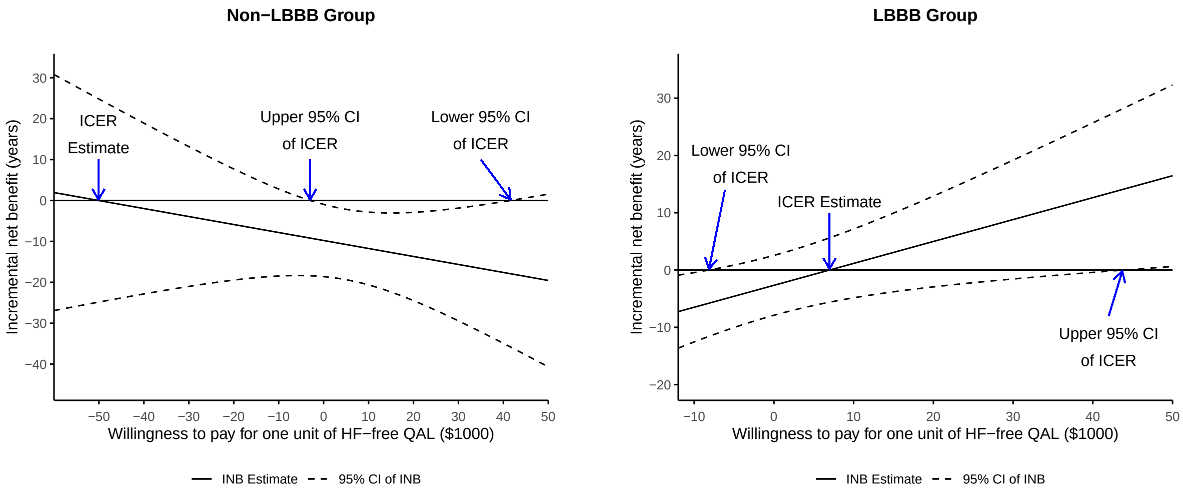

Web Figure 2: Estimated INBs and 95% CIs using HF-free QAL as the effectiveness measure for LBBB and non-LBBB groups in the MADIT-CRT study, limited to a 4-year time horizon. The solid lines denote the INB estimates as a function of WTP; the dashed lines indicate the corresponding CI limits for INBs obtained by our method. The horizontal intercepts represent the ICER estimates and their corresponding CI limits, calculated using our method as described in the main text.

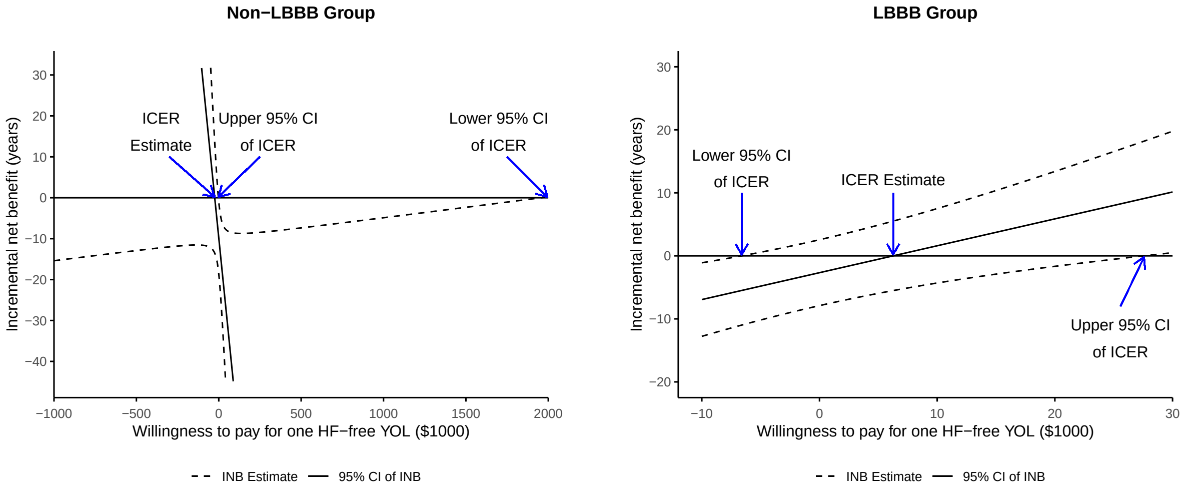

Web Figure 3: Estimated INBs and 95% CIs using HF-free YOL as the effectiveness measure for LBBB and non-LBBB groups in the MADIT-CRT study, limited to a 4-year time horizon. The solid lines denote the INB estimates as a function of WTP; the dashed lines indicate the corresponding CI limits for INBs obtained by our method. The horizontal intercepts represent the ICER estimates and their corresponding CI limits, calculated using our method as described in the main text.

C.2: Additional results for INB using data with different censoring times

This subsection presents the additional numerical results for the estimated INB using data with different censoring times in both simulation (main text Section 3.2) and the MADIT-II example (main text Section 4.2). Web Table 13 provides summaries of the simulation results based on 2000 simulation runs, considering various sample sizes and WTP values. The measurements of effectiveness considered are QAL and YOL, respectively. Web Figure 4 presents the INB plot for MADIT-II study using YOL as the effectiveness measure.

Web Table 13: Summary of estimated INBs (in \$1000) using QAL or YOL as the effectiveness measure with different WTP values (in \$1000), based on data with different censoring times from 2000 simulations.

				QAL		YOL	
n	WTP	Subgroup U	Method	Bias (SEE, ESE)	CP	Bias (SEE, ESE)	CP
400	10	0	SW	0.11 (4.14, 4.07)	0.943	0.16 (5.22, 5.14)	0.943
			IMP	0.09 (4.22, 4.23)	0.949	0.15 (5.21, 5.13)	0.946
		1	SW	-0.18 (4.22, 4.22)	0.951	-0.20 (5.23, 5.21)	0.948
			IMP	-0.13 (4.56, 4.61)	0.953	-0.18 (5.22, 5.20)	0.949
	20	0	SW	0.23 (8.75, 8.64)	0.946	0.34 (10.92, 10.77)	0.945
			IMP	0.21 (9.00, 9.02)	0.949	0.32 (10.92, 10.77)	0.944
		1	SW	-0.36 (8.64, 8.63)	0.950	-0.40 (10.67, 10.65)	0.949
			IMP	-0.27 (9.36, 9.47)	0.952	-0.38 (10.66, 10.63)	0.949
800	10	0	SW	0.03 (2.83, 2.88)	0.957	0.07 (3.58, 3.63)	0.957
			IMP	0.01 (2.93, 2.99)	0.955	0.07 (3.58, 3.63)	0.957
		1	SW	0.02 (2.95, 2.98)	0.953	0.01 (3.64, 3.68)	0.952
			IMP	0.02 (3.22, 3.25)	0.954	0.02 (3.64, 3.67)	0.952
	20	0	SW	0.06 (6.00, 6.11)	0.957	0.14 (7.51, 7.62)	0.954
			IMP	0.02 (6.26, 6.37)	0.956	0.14 (7.51, 7.61)	0.955
		1	SW	0.04 (6.05, 6.09)	0.951	0.02 (7.43, 7.52)	0.954
			IMP	0.03 (6.60, 6.68)	0.954	0.03 (7.44, 7.51)	0.953
1200	10	0	SW	0.05 (2.38, 2.35)	0.944	0.07 (3.02, 2.96)	0.943
			IMP	0.03 (2.46, 2.44)	0.946	0.08 (3.03, 2.96)	0.941
		1	SW	-0.08 (2.42, 2.43)	0.946	-0.13 (2.98, 3.01)	0.949
			IMP	-0.08 (2.65, 2.66)	0.945	-0.13 (2.98, 3.00)	0.949
	20	0	SW	0.11 (5.06, 4.98)	0.944	0.15 (6.34, 6.22)	0.943
			IMP	0.06 (5.26, 5.20)	0.943	0.16 (6.35, 6.21)	0.941
		1	SW	-0.17 (4.96, 4.98)	0.946	-0.26 (6.10, 6.15)	0.947
			IMP	-0.17 (5.45, 5.46)	0.946	-0.26 (6.09, 6.14)	0.947

Notes: WTP is decision maker's willingness-to-pay for an additional unit of effectiveness; Bias represents the absolute difference between the mean estimate and the true value; SEE indicates the empirical standard error of the estimates; ESE is the mean of the estimated standard errors; CP is the proportion containing the true INB within the 95% CI; SW denotes the simple weighted estimator for early-censored costs, QAL, and YOL; IMP refers to the improved estimator for early-censored costs and QAL only.

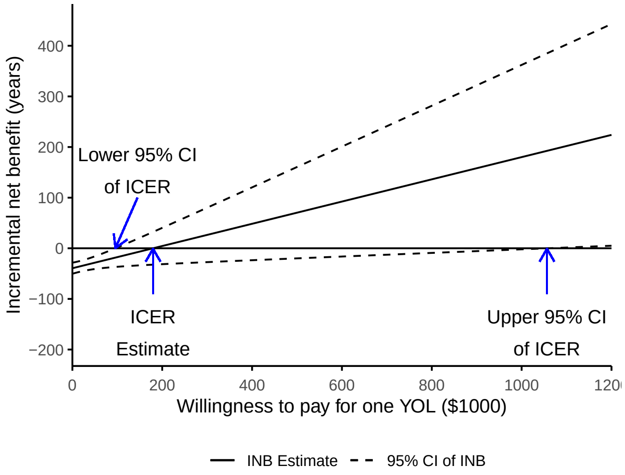

Web Figure 4: Estimated covariate-adjusted INB and 95% CI using YOL as the effectiveness measure in the MADIT-II study, limited to a 3.5-year time horizon. The solid line denotes the INB estimate as a function of WTP; the dashed lines indicate the corresponding CI limits for INB obtained by our method. The horizontal intercepts represent the ICER estimate and its corresponding CI limits, calculated using our method as described in the main text.

Web Appendix D: Additional numerical results for regression coefficient estimates

D.1: Additional results for regression coefficient estimates using data with different terminating events

This subsection reports additional numerical results for the regression coefficient estimators based on simulated data with different terminating events (main text Section 3.1). Web Table 14 and Web Table 15 present the simulation results of the estimated regression coefficients in Equation (1) of the main text, used to obtain ICERs (described in Section 2.3.3) based on 2,000 simulations under heavy and light censoring, respectively. Both the SW and IMP methods yield minimal and comparable bias in estimating costs and effectiveness. The ESEs of all estimators are close to the SEE, and they decrease with increasing sample size from 400 to 1200. As noted in Wang and Zhao (2007), the IMP estimator is not guaranteed to

be more efficient than the SW estimator, as the estimation of an unknown term introduces further variability. For HF-free QAL, the IMP estimator exhibits greater efficiency than the SW estimator, as reflected by smaller standard errors and improved CPs, especially under heavy censoring. For cost, the IMP method demonstrates smaller bias and improved CPs with small sample size ($n = 400$) under heavy censoring. However, for data with large samples or light censoring, the IMP method does not show superiority to the SW estimators for cost.

Web Table 14: Summary of estimated regression coefficients based on data with different terminating events under heavy censoring from 2000 simulations.

Variable	n	Estimator	SW		IMP	
			Bias (SEE, ESE)	CP	Bias (SEE, ESE)	CP
Cost ($1000)	400	β_0	-0.04 (0.53, 0.51)	0.921	0.00 (0.56, 0.54)	0.935
		β_Z	0.01 (0.68, 0.65)	0.934	-0.01 (0.72, 0.70)	0.941
		β_A	0.00 (1.01, 0.98)	0.933	0.01 (1.00, 0.99)	0.943
		β_{A*Z}	0.01 (1.13, 1.08)	0.932	-0.01 (1.14, 1.11)	0.942
	800	β_0	-0.02 (0.37, 0.36)	0.939	-0.00 (0.39, 0.39)	0.946
		β_Z	-0.00 (0.47, 0.46)	0.936	-0.01 (0.50, 0.50)	0.946
		β_A	-0.00 (0.70, 0.70)	0.941	-0.01 (0.69, 0.70)	0.958
		β_{A*Z}	0.01 (0.79, 0.77)	0.938	0.02 (0.79, 0.79)	0.950
	1200	β_0	-0.03 (0.29, 0.29)	0.946	-0.02 (0.31, 0.31)	0.945
		β_Z	0.02 (0.38, 0.38)	0.952	0.02 (0.40, 0.41)	0.950
		β_A	0.00 (0.58, 0.56)	0.941	0.02 (0.58, 0.57)	0.943
		β_{A*Z}	0.00 (0.64, 0.63)	0.945	-0.01 (0.66, 0.64)	0.942
HF-free QAL	400	β_0	-0.01 (0.30, 0.29)	0.893	0.00 (0.24, 0.23)	0.927
		β_Z	0.00 (0.37, 0.36)	0.922	-0.00 (0.30, 0.29)	0.941
		β_A	0.00 (0.38, 0.37)	0.925	-0.00 (0.31, 0.30)	0.947
		β_{A*Z}	-0.02 (0.60, 0.58)	0.945	-0.01 (0.60, 0.59)	0.941
	800	β_0	-0.00 (0.21, 0.21)	0.934	-0.00 (0.16, 0.17)	0.946
		β_Z	-0.00 (0.26, 0.26)	0.946	-0.00 (0.20, 0.21)	0.950
		β_A	-0.00 (0.27, 0.26)	0.944	-0.01 (0.22, 0.22)	0.952
		β_{A*Z}	0.01 (0.42, 0.42)	0.946	0.02 (0.42, 0.42)	0.948
	1200	β_0	-0.01 (0.17, 0.17)	0.932	-0.00 (0.14, 0.14)	0.941
		β_Z	0.01 (0.21, 0.21)	0.945	0.00 (0.17, 0.17)	0.947
		β_A	0.01 (0.22, 0.22)	0.941	0.01 (0.18, 0.18)	0.947
		β_{A*Z}	-0.01 (0.35, 0.34)	0.943	0.00 (0.35, 0.34)	0.938
HF-free YOL	400	β_0	-0.02 (0.46, 0.44)	0.894	-	-
		β_Z	0.01 (0.57, 0.56)	0.922	-	-
		β_A	0.01 (0.52, 0.51)	0.914	-	-
		β_{A*Z}	-0.02 (0.74, 0.73)	0.941	-	-
	800	β_0	-0.01 (0.32, 0.32)	0.933	-	-
		β_Z	-0.00 (0.40, 0.40)	0.946	-	-
		β_A	-0.00 (0.37, 0.36)	0.935	-	-
		β_{A*Z}	0.01 (0.52, 0.52)	0.947	-	-
	1200	β_0	-0.01 (0.26, 0.26)	0.931	-	-
		β_Z	0.01 (0.33, 0.33)	0.947	-	-
		β_A	0.01 (0.30, 0.30)	0.939	-	-
		β_{A*Z}	-0.01 (0.43, 0.42)	0.944	-	-

Notes: Bias represents the absolute difference between the mean estimate and the true value; SEE indicates the empirical standard error of the estimates; ESE is the mean of the estimated standard errors; CP is the proportion containing the true value within the 95% CI; SW denotes the simple weighted estimator for costs, HF-free QAL, and HF-free YOL; IMP refers to the improved estimator for both costs and HF-free QAL.

Web Table 15: Summary of estimated regression coefficients based on data with different terminating events under light censoring from 2000 simulations.

Variable	n	Estimator	SW		IMP	
			Bias (SEE, ESE)	CP	Bias (SEE, ESE)	CP
Cost ($1000)	400	β_0	-0.00 (0.39, 0.39)	0.949	0.01 (0.44, 0.43)	0.941
		β_Z	-0.01 (0.49, 0.48)	0.946	-0.01 (0.56, 0.55)	0.939
		β_A	0.00 (0.73, 0.71)	0.944	0.00 (0.80, 0.78)	0.938
		β_{A*Z}	-0.00 (0.81, 0.79)	0.941	-0.01 (0.90, 0.88)	0.939
	800	β_0	-0.00 (0.28, 0.27)	0.943	0.00 (0.31, 0.31)	0.950
		β_Z	-0.01 (0.35, 0.34)	0.947	-0.00 (0.39, 0.39)	0.949
		β_A	-0.01 (0.51, 0.51)	0.946	-0.02 (0.55, 0.55)	0.957
		β_{A*Z}	0.02 (0.57, 0.56)	0.944	0.02 (0.62, 0.62)	0.953
	1200	β_0	-0.01 (0.21, 0.22)	0.955	-0.01 (0.24, 0.25)	0.960
		β_Z	0.01 (0.27, 0.28)	0.959	0.01 (0.30, 0.32)	0.959
		β_A	0.01 (0.41, 0.41)	0.954	0.01 (0.45, 0.45)	0.951
		β_{A*Z}	-0.01 (0.46, 0.46)	0.956	-0.01 (0.51, 0.51)	0.947
HF-free QAL	400	β_0	-0.00 (0.22, 0.22)	0.934	0.00 (0.21, 0.21)	0.937
		β_Z	-0.01 (0.27, 0.27)	0.939	-0.01 (0.26, 0.26)	0.942
		β_A	-0.00 (0.29, 0.29)	0.944	-0.00 (0.28, 0.27)	0.944
		β_{A*Z}	-0.01 (0.50, 0.49)	0.949	-0.01 (0.52, 0.51)	0.943
	800	β_0	-0.00 (0.15, 0.15)	0.942	0.00 (0.15, 0.15)	0.945
		β_Z	0.00 (0.19, 0.19)	0.953	-0.00 (0.18, 0.18)	0.947
		β_A	-0.00 (0.21, 0.20)	0.944	-0.01 (0.19, 0.19)	0.948
		β_{A*Z}	0.02 (0.35, 0.35)	0.948	0.02 (0.37, 0.36)	0.945
	1200	β_0	-0.00 (0.12, 0.13)	0.951	-0.00 (0.12, 0.12)	0.953
		β_Z	0.00 (0.16, 0.16)	0.959	0.00 (0.15, 0.15)	0.956
		β_A	0.00 (0.16, 0.17)	0.946	0.00 (0.16, 0.16)	0.951
		β_{A*Z}	0.00 (0.29, 0.28)	0.945	0.01 (0.30, 0.30)	0.940
HF-free YOL	400	β_0	-0.00 (0.34, 0.33)	0.934	-	-
		β_Z	-0.01 (0.42, 0.42)	0.939	-	-
		β_A	-0.00 (0.39, 0.39)	0.940	-	-
		β_{A*Z}	-0.00 (0.61, 0.60)	0.951	-	-
	800	β_0	-0.00 (0.24, 0.24)	0.944	-	-
		β_Z	0.00 (0.30, 0.30)	0.953	-	-
		β_A	-0.00 (0.28, 0.28)	0.941	-	-
		β_{A*Z}	0.02 (0.43, 0.42)	0.945	-	-
	1200	β_0	-0.00 (0.19, 0.19)	0.950	-	-
		β_Z	0.00 (0.24, 0.24)	0.960	-	-
		β_A	0.00 (0.22, 0.22)	0.948	-	-
		β_{A*Z}	0.00 (0.35, 0.35)	0.943	-	-

Notes: Bias represents the absolute difference between the mean estimate and the true value; SEE indicates the empirical standard error of the estimates; ESE is the mean of the estimated standard errors; CP is the proportion containing the true value within the 95% CI; SW denotes the simple weighted estimator for costs, HF-free QAL, and HF-free YOL; IMP refers to the improved estimator for both costs and HF-free QAL.

D.2: Additional results for regression coefficient estimates using data with different censoring times

This subsection presents the additional numerical results for the regression coefficient estimators based on simulated data with different censoring times (main text Section 3.2). Web Table 16 provides summaries of the simulation results of the estimated regression coefficients based on 2000 simulation runs. For small sample size ($n = 400$), the IMP method yields higher CPs for both costs and QAL than the SW method. With large sample size, the CPs of both methods are close to nominal level. In addition, the IMP method does not show improved efficiency. These results align with Table 2 in the main text, where the IMP method provides better CPs but slightly wider CIs.

Web Table 16: Summary of estimated regression coefficients based on data with different censoring times from 2000 simulations.

Variable	n	Estimator	SW		IMP	
			Bias (SEE, ESE)	CP	Bias (SEE, ESE)	CP
Cost ($1000)	400	β_0	-0.05 (0.53, 0.52)	0.932	-0.02 (0.58, 0.58)	0.947
		β_Z	0.04 (0.67, 0.67)	0.949	0.04 (0.76, 0.76)	0.955
		β_A	-0.02 (1.03, 1.00)	0.932	0.00 (1.06, 1.06)	0.946
		β_{A*Z}	0.02 (1.13, 1.11)	0.939	-0.03 (1.20, 1.20)	0.945
	800	β_0	-0.03 (0.37, 0.37)	0.943	-0.01 (0.41, 0.41)	0.949
		β_Z	0.02 (0.47, 0.48)	0.942	0.00 (0.53, 0.54)	0.950
		β_A	0.02 (0.72, 0.71)	0.944	0.01 (0.77, 0.75)	0.935
		β_{A*Z}	-0.01 (0.80, 0.79)	0.940	-0.01 (0.87, 0.85)	0.944
	1200	β_0	-0.01 (0.31, 0.30)	0.941	-0.00 (0.34, 0.34)	0.948
		β_Z	0.01 (0.40, 0.39)	0.942	0.01 (0.44, 0.44)	0.952
		β_A	0.01 (0.60, 0.58)	0.944	0.01 (0.63, 0.61)	0.952
		β_{A*Z}	-0.02 (0.67, 0.64)	0.941	-0.02 (0.70, 0.69)	0.951
QAL	400	β_0	-0.01 (0.26, 0.26)	0.944	-0.01 (0.27, 0.27)	0.952
		β_Z	0.01 (0.36, 0.36)	0.950	0.01 (0.38, 0.38)	0.952
		β_A	-0.01 (0.47, 0.46)	0.945	-0.01 (0.48, 0.48)	0.947
		β_{A*Z}	-0.01 (0.65, 0.64)	0.937	-0.01 (0.69, 0.69)	0.942
	800	β_0	-0.01 (0.18, 0.18)	0.950	-0.00 (0.19, 0.19)	0.953
		β_Z	0.01 (0.25, 0.26)	0.953	0.01 (0.26, 0.27)	0.959
		β_A	0.01 (0.33, 0.33)	0.948	0.01 (0.35, 0.34)	0.947
		β_{A*Z}	-0.01 (0.44, 0.45)	0.951	-0.01 (0.47, 0.49)	0.953
	1200	β_0	-0.00 (0.15, 0.15)	0.947	-0.00 (0.16, 0.16)	0.942
		β_Z	0.01 (0.21, 0.21)	0.948	0.01 (0.22, 0.22)	0.953
		β_A	0.01 (0.27, 0.27)	0.949	0.01 (0.28, 0.28)	0.948
		β_{A*Z}	-0.01 (0.37, 0.37)	0.945	-0.01 (0.40, 0.40)	0.943
YOL	400	β_0	-0.02 (0.40, 0.40)	0.943	-	-
		β_Z	0.02 (0.55, 0.56)	0.950	-	-
		β_A	-0.01 (0.58, 0.57)	0.946	-	-
		β_{A*Z}	-0.02 (0.80, 0.79)	0.940	-	-
	800	β_0	-0.01 (0.28, 0.28)	0.950	-	-
		β_Z	0.01 (0.39, 0.39)	0.953	-	-
		β_A	0.01 (0.40, 0.40)	0.947	-	-
		β_{A*Z}	-0.02 (0.54, 0.56)	0.950	-	-
	1200	β_0	-0.01 (0.23, 0.23)	0.946	-	-
		β_Z	0.01 (0.32, 0.32)	0.949	-	-
		β_A	0.01 (0.33, 0.33)	0.950	-	-
		β_{A*Z}	-0.02 (0.45, 0.45)	0.946	-	-

Notes: Bias represents the absolute difference between the mean estimate and the true value; SEE indicates the empirical standard error of the estimates; ESE is the mean of the estimated standard errors; CP is the proportion containing the true value within the 95% CI; SW denotes the simple weighted estimator for costs, QAL, and YOL; IMP refers to the improved estimator for both costs and QAL.

Web Appendix E: Derivation of the true values of mean effectiveness and mean costs in simulations

Let survival time T be generated from an exponential distribution with rate parameter $\lambda_0 = 1/exp(\boldsymbol{b}'\boldsymbol{Z})$, where $\boldsymbol{b}$ is the vector of coefficients and $\boldsymbol{Z} = (1, \boldsymbol{U}, A, A\boldsymbol{U})'$. T is then truncated at time L by $T^L = \min(T, L)$. From Chen and Hoch (2022), $E(T^L|A, \boldsymbol{U}) = \frac{1}{\lambda_0}(1 - e^{-\lambda_0 L})$. Denote M_{diag} as the diagnostic costs, M_{ann} as the fixed annual costs, M_{ran} as the random annual costs, and M_{term} as the termination costs that assumed to be evenly distributed within the last year of survival. The true mean cost within time L given A and $\boldsymbol{U}$ is

$$
\begin{aligned}
E\{M(T^L)|A, \boldsymbol{U}\} &= E\{M_{diag}|A, \boldsymbol{U}\} + E\{(M_{ann} + M_{ran}) \cdot T^L|A, \boldsymbol{U}\} \\
&\quad + E\{M_{term} \cdot I(T \leq L)|A, \boldsymbol{U}\} \\
&\quad + E\{(L + 1 - T) \cdot M_{term} \cdot I(L < T \leq L + 1)|A, \boldsymbol{U}\} \\
&= E\{M_{diag}|A, \boldsymbol{U}\} + E\{T^L|A, \boldsymbol{U}\} \cdot E\{(M_{ann} + M_{ran})|A, \boldsymbol{U}\} \\
&\quad + P(T \leq L|A, \boldsymbol{U}) \cdot E\{M_{term}|A, \boldsymbol{U}\} \\
&\quad + E\{(L + 1 - T) \cdot I(L < T \leq L + 1)|A, \boldsymbol{U}\} \cdot E\{M_{term}|A, \boldsymbol{U}\} \\
&= E\{M_{diag}|A, \boldsymbol{U}\} + \frac{1}{\lambda_0}(1 - e^{-\lambda_0 L}) \cdot E\{M_{ann}|A, \boldsymbol{U}\} \\
&\quad + \frac{1}{\lambda_0}(1 - e^{-\lambda_0 L}) \cdot E\{M_{ran}|A, \boldsymbol{U}\} + (1 - e^{-\lambda_0 L}) \cdot E\{M_{term}|A, \boldsymbol{U}\} \\
&\quad + (\int_L^{L+1} (L + 1 - T) \cdot f(T)\, dT) \cdot E\{M_{term}|A, \boldsymbol{U}\} \\
&= E\{M_{diag}|A, \boldsymbol{U}\} + \frac{1}{\lambda_0}(1 - e^{-\lambda_0 L}) \cdot E\{M_{ann}|A, \boldsymbol{U}\} \\
&\quad + \frac{1}{\lambda_0}(1 - e^{-\lambda_0 L}) \cdot E\{M_{ran}|A, \boldsymbol{U}\} + (1 - e^{-\lambda_0 L}) \cdot E\{M_{term}|A, \boldsymbol{U}\} \\
&\quad + (e^{-\lambda_0 L} + \frac{1}{\lambda_0}(e^{-\lambda_0(L+1)} - e^{-\lambda_0 L}))E\{M_{term}|A, \boldsymbol{U}\} \\
&= E\{M_{diag}|A, \boldsymbol{U}\} + \frac{1}{\lambda_0}(1 - e^{-\lambda_0 L}) \cdot E\{M_{ann}|A, \boldsymbol{U}\} \\
&\quad + \frac{1}{\lambda_0}(1 - e^{-\lambda_0 L}) \cdot E\{M_{ran}|A, \boldsymbol{U}\} \\
&\quad + (1 + \frac{1}{\lambda_0}(e^{-\lambda_0(L+1)} - e^{-\lambda_0 L}))E\{M_{term}|A, \boldsymbol{U}\}.
\end{aligned}
$$

References

Bang, H. and Tsiatis, A. A. (2000). Estimating medical costs with censored data. *Biometrika* **87,** 329–343.

Chen, S. and Hoch, J. S. (2022). Net-benefit regression with censored cost-effectiveness data from randomized or observational studies. *Statistics in Medicine* **41,** 3958–3974.

Chen, S. and Zhao, H. (2013). Estimating incremental cost-effectiveness ratios and their confidence intervals with different terminating events for survival time and costs. *Biostatistics* **14,** 422–432.

Lin, D. Y. (2000). Linear regression analysis of censored medical costs. *Biostatistics* **1,** 35–47.

Wang, H. and Zhao, H. (2006). Estimating incremental cost-effectiveness ratios and their confidence intervals with differentially censored data. *Biometrics* **62,** 570–575.

Wang, H. and Zhao, H. (2007). Regression analysis of mean quality-adjusted lifetime with censored data. *Biostatistics* **8,** 368–382.

Wang, H. and Zhao, H. (2008). A study on confidence intervals for incremental cost-effectiveness ratios. *Biometrical Journal* **50,** 505–514.

Willan, A. R., Lin, D. Y., and Manca, A. (2005). Regression methods for cost-effectiveness analysis with censored data. *Statistics in Medicine* **15,** 131–145.
